# Genetic diversity analysis in Chinese miniature pigs using swine leukocyte antigen complex microsatellites

**DOI:** 10.5713/ab.20.0637

**Published:** 2021-02-16

**Authors:** Jinhua Wu, Ronghui Liu, Hua Li, Hui Yu, Yalan Yang

**Affiliations:** 1 Guangdong Provincial Key Laboratory of Animal Molecular Design and Precise Breeding, Foshan University, Foshan 528225, China; 2 Agriculture and Rural Affairs Committee of Kaizhou District, Chongqing Municipality, Chongqing 405400, China

**Keywords:** Diversity, Haplotypes, Microsatellite, Miniature Pigs, Swine Leukocyte Antigen (SLA)

## Abstract

**Objective:**

The swine leukocyte antigen (SLA) gene group, which is closely linked and highly polymorphic, has important biomedical significance in the protection and utilization of germplasm resources. However, genetic polymorphism analyses of SLA microsatellite markers in Chinese miniature pigs are limited.

**Methods:**

Eighteen pairs of microsatellite primers were used to amplify the SLA regions of seven miniature pig breeds and three wild boar breeds (n = 346) from different regions of China. The indexes of genetic polymorphism, including expected heterozygosity (He), polymorphic information content (PIC), and haplotype, were analyzed. The genetic differentiation coefficient (Fst) and neighbor-joining methods were used for cluster analysis of the breeds.

**Results:**

In miniature pigs, the SLA I region had the highest numbers of polymorphisms, followed by the SLA II and SLA III regions; the region near the centromere had the lowest number of polymorphisms. Among the seven miniature pig breeds, Diannan small-ear pigs had the highest genetic diversity (PIC value = 0.6396), whereas the genetic diversity of the Hebao pig was the lowest (PIC value = 0.4330). The Fst values in the Mingguang small-ear, Diannan small-ear, and Yunnan wild boars were less than 0.05. According to phylogenetic cluster analysis, the South-China-type miniature pigs clustered into one group, among which Mingguang small-ear pigs clustered with Diannan small-ear pigs. Haplotype analysis revealed that the SLA I, II, and III regions could be constructed into 13, 7, and 11 common haplotypes, respectively.

**Conclusion:**

This study validates the high genetic diversity of the Chinese miniature pig. Mingguang small-ear pigs have close kinship with Diannan small-ear pigs, implying that they may have similar genetic backgrounds and originate from the same population. This study also provides a foundation for genetic breeding, genetic resource protection, and classification of Chinese miniature pigs.

## INTRODUCTION

Developing suitable animal models is a crucial prerequisite for the development of safe preclinical protocols in biomedical research, which allows for human-related validation of valuable research information gathered from experimentation with lower mammals. Miniature pigs can be used in long-term experiments owing to their long lifespan; they can also be easily bred and handled because of their small size and short reproduction cycle. Miniature pigs have become promising donor animals for xenotransplantation because of their anatomical and physiological characteristics that are highly similar to those of humans [1,2]. Miniature pigs are abundant in China and are mainly distributed in the southwestern and southern regions. Recent studies have shown that some breeds of Chinese miniature pigs, such as Diannan small-ear, Wuzhishan, and Bama, may be candidate donor sources for human xenotransplantation [2]. However, with the gradual expansion of the animal product consumption market and the invasion of commercial Western pigs, some breeds are almost endangered [3]. Therefore, it is necessary to protect the germplasm resources of Chinese miniature pigs while making rational use of them.

The swine major histocompatibility complex (MHC) is referred to as the swine leukocyte antigen (SLA), which has been mapped to the *Sus scrofa* (SSC) chromosome 7 region spanning the centromere [4]. SLA consists of three regions: the class I and III regions map to pig chromosome 7p1.1, and the class II region maps to 7q1.1. SLA, a closely linked and highly polymorphic gene group exhibiting uniform distributions of many microsatellites, which has been widely used to detect genetic diversity, is closely related to the immune system, breeding, reproductive traits, and xenotransplantation [4,5].

To detect genetic diversity and differentiate between breeds, several molecular-marker techniques are available, including restriction fragment length polymorphisms, variable numbers of tandem repeats, denaturing gradient gel electrophoresis, single-strand conformational polymorphism, random amplified polymorphic DNA, and microsatellite markers (MS) [6]. MS are short tandem repeats, short sequence repeats, or sequence tagged microsatellite sites that contain repetitive sequences composed of 2 to 6 nucleotides [7]. Microsatellites have been proposed as the best markers for evaluating the genetic diversity of domestic animals because of their abundance, even distribution in the genome, high levels of polymorphism, and ease of genotyping [7]. Charoensook et al [8] used 26 MS to study the genetic diversity and carry out phylogenetic analyses of Thai native pigs. MS data showed that Thai native pigs had high genetic diversity and were closely related to Chinese pigs. MS have also been used to analyze the genetic diversity of some Chinese miniature pig breeds [9].

However, the genetic diversity of the SLA region of Chinese miniature pigs remains mostly unknown. To understand the genetic diversity of Chinese miniature pigs and compare the genetic differences of miniature pigs in different regions of China, we determined the genetic polymorphisms of MSs of SLA in seven Chinese miniature pig breeds and three wild boar breeds. This study is not only beneficial for the protection and utilization of miniature pig resources but also provides information for pig disease-resistance breeding and human medical models.

## MATERIALS AND METHODS

### Animal care

The study was approved by the Animal Care Committee of Foshan University (Foshan, China). All animal experiments were performed in accordance with the national guidelines for animal welfare.

### Animals and tissue sampling

**Figure 1:**
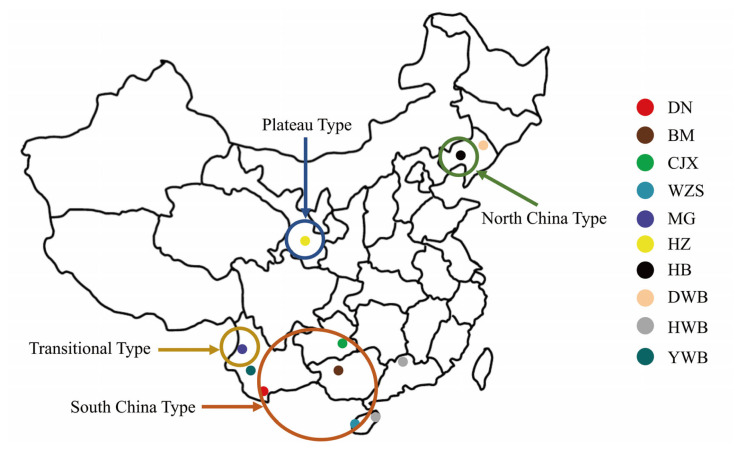
Geographical distribution of each breed. DN, Diannan small-ear pig; BM, Bama miniature pig; CJX, Congjiang Xiang pig; WZS, Wuzhishan pig; MG, Mingguang small-ear pig; HZ, Hezuo pig; HB, Hebao pig; DWB, Dongbei wild boar; YWB, Yunnan wild boar; HWB, Huanan wild boar.

A total of 346 animals from seven Chinese miniature pig breeds and three wild boar breeds were sampled for polymorphism detection. According to their geographical distribution, the seven Chinese miniature pig breeds were classified into four types: South-China-type, transitional-type, plateau-type, and North-China-type. The transitional-type, such as the Mingguang small-ear pig, refers to those whose habitat altitude and phenotypic characteristics are between plateau-type and the South-China-type miniature pigs. More details about the breeds, abbreviations, numbers, locations, and types are given in Table 1 and Figure 1. Before sampling, each pig was restrained safely, and the right ear was sterilized. Ear tissue of about 2.0 cm×0.5 cm was quickly cut out from the outer edge of the ear with an ear clamp. The sample for each pig was quickly placed in a sterile tube and stored at −80°C for later use.

### Polymerase chain reaction amplification and product detection

The genomic DNA of each sample was extracted from the frozen ear tissue using a standard phenol/chloroform extraction method. The extracted DNA was stored at −20°C. According to a previous study [10], 18 microsatellite loci in the SLA region were selected, including four loci near the SLA classic class I gene and two loci near the non-classic class I gene, nine loci in the whole SLA class II region, and three highly polymorphic loci in the SLA class III region (Supplementary Figure S1). The primers were synthesized by Sangon Biotech (Shanghai, China). The forward primers were fluorescently labeled with fluorescein amidite at their 5′-ends (Supplementary Table S1). The polymerase chain reaction (PCR) reaction was a 10-μL volume containing 1.0 μL of 10× buffer (containing Mg^2+^; Takara, Dalian, China), 0.4 μL dNTPs (2.5 mM), 0.12 μL each of forward and reverse primers (10 pmol/μL), 0.1 μL of Taq DNA polymerase (5 U/μL, Takara, China), 0.8 μL of DNA template (50 ng/μL), and double-distilled water to 10 μL. The reaction conditions were as follows: initial denaturation at 94.0°C for 5 min; 35 cycles of 30 s at 94°C, 30 s annealing at 55 to 60°C, and 30 s extension at 72°C. The final extension step at 72°C was prolonged for 7 min and the thermocycler held at 4.0°C. The PCR products were identified using 1.5% agarose gel electrophoresis. Depending on the intensity of the band on the gel, PCR products were diluted 1:10 to 1:50 in double-distilled water. After dilution, a mixture of 1 μL PCR products, 8.8 μL formamide, and 0.2 μL GeneScan Liz-500 Size Standard (Applied Biosystems, Foster City, CA, USA) was prepared and denatured at 95 °C for 5 min followed by heat shock and chilling on ice at 0°C for 5 min. The mixtures were sequenced using an ABI3730 DNA analyzer (Applied Biosystems, USA).

### Data transformations and statistical analysis

The collected raw data were analyzed using GeneMapper software (version 4.0; Applied Biosystems, USA) and corrected using FlexiBin version 2 [11]. The expected heterozygosity (He) and polymorphic information content (PIC) were calculated using the Microsatellite Toolkit software [12]. The higher the He value, the richer the genetic diversity of the population. Deviations from the Hardy–Weinberg equilibrium (HWE) were calculated using GENEPOP version 4.2 [13]. Genetic differentiation coefficient (Fst) values of genetic differentiation were calculated using Arlequin version 3.5.2, and haplotype frequencies were obtained using the expectation-maximization algorithm [14]. According to the criterion defined by Wright [15], we defined genetic differentiation as low for Fst<0.05, moderate for 0.05<Fst<0.15, high for 0.15 <Fst<0.25, and very high for Fst>0.25. Genetic distance analysis was calculated using the Nei’s genetic distance (DA) and Cavalli-Sforza Chord distance (Dc) methods with MSA 4.05 [16]; genetic relationship analysis was constructed using the neighbor-joining (NJ) method with MEGA 7 [17].

## RESULTS

### Microsatellite polymorphisms in the swine leukocyte antigen regions

**Figure 2:**
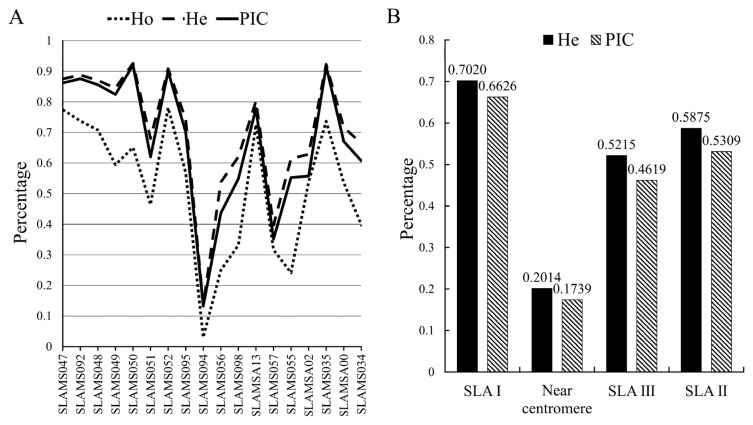
Analysis of microsatellite polymorphism in SLA region. (A) The polymorphism of the SLA microsatellite in the whole population; the ordinate indicates the calculated value of each parameter and the abscissa indicates the microsatellite locus. (B) Polymorphisms of microsatellites in different regions of SLA; the abscissa indicates the different regions of SLA. SLA, swine leukocyte antigen; Ho, observed heterozygosity; He, expected heterozygosity; PIC, polymorphism information content.

Based on the MS data analysis, we found that different SLA loci in Chinese miniature pigs had different genetic polymorphisms (Figure 2A). *SLAMS050* had the highest polymorphism level (He value = 0.9260, PIC value = 0.9202), whereas *SLAMS094* had the lowest polymorphism level (He value = 0.1367, PIC value = 0.1324), and the other 16 microsatellites had relatively higher polymorphism levels. The order of numbers of microsatellite polymorphisms in the SLA region was SLA I>SLA II>SLA III. As expected, the region near the centromere had low levels of polymorphism (Figure 2B).

### Intrapopulation genetic diversity

When we compared the genetic diversity within breeds, we found that the He of Chinese miniature pigs was over 0.62, and the PIC was above 0.57, indicating that Chinese miniature pigs have high intra-breed genetic variation (Table 2). Transitional miniature pigs (Mingguang small-ear pigs) had the highest genetic diversity, followed by the South-China, plateau, and North-China types. The He and PIC of South-China-type miniature pigs were significantly higher than those of North-China-type miniature pigs (p<0.05). However, there was no significant difference between South-China-type miniature pigs and plateau-type miniature pigs (p> 0.05). Wild boars also have rich genetic diversity. Although the genetic diversity of wild boars was higher than that of Chinese miniature pigs, there was no significant difference between them (p>0.05). Of the seven miniature pig breeds, Diannan small-ear pigs exhibited the highest genetic diversity, followed by Mingguang small-ear pigs, Wuzhishan pigs, Bama miniature pigs, Hezuo pigs, Congjiang Xiang pigs, and Hebao pigs (Supplementary Table S2). The number of loci that deviated from the HWE in each breed ranged from three (Dongbei wild boar) to 13 (Mingguang small-ear pig and Diannan small-ear pig) (Supplementary Table S2).

### Interpopulation genetic diversity

**Figure 3:**
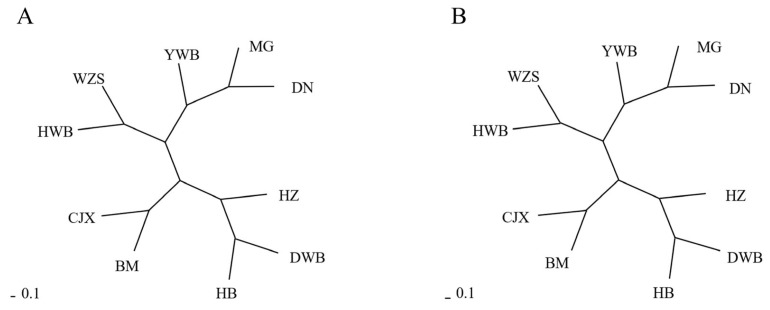
The genetic distance among ten pig breeds. (A) Neighbor-joining tree using the Dc distance. (B) Neighbor-joining tree using the DA distance. DN, Diannan small-ear pig; BM, Bama miniature pig; CJX, Congjiang Xiang pig; WZS, Wuzhishan pig; MG, Mingguang small-ear pig; HZ, Hezuo pig; HB, Hebao pig; DWB, Dongbei wild boar; YWB, Yunnan wild boar; HWB, Huanan wild boar.

We calculated the Fst values of the seven Chinese miniature pig breeds and three wild boar breeds (Table 3). The results showed that the Fst values of the Mingguang small-ear pigs, Diannan small-ear pigs, and Yunnan wild boar were all less than 0.05, indicating a low degree of differentiation among the three breeds. Except for Hezuo pigs, the Fst values of Hebao pigs and other pig breeds were all greater than 0.15, indicating that the other breeds were highly differentiated from Hebao pigs. The genetic distance between Chinese miniature pigs and wild boars was calculated by the DA and Dc methods with MSA 4.05 (Supplementary Table S3). The result of the NJ tree constructed using the DA method (Figure 3A) was consistent with those constructed using the Dc method (Figure 3B). The NJ tree revealed that seven Chinese miniature pig breeds and three wild boar breeds were divided into four clades. The Mingguang small-ear pig clustered with the Diannan small-ear pig and Yunnan wild boar. Wuzhishan pigs and Huanan wild boar were clustered. Dongbei wild boar, the Hebao pig, and the Hezuo pig were clustered together. Then, the Bama miniature pig was clustered with the Congjiang Xiang pig. Overall, the South-China-type miniature pigs were clustered into one group. The transitional miniature pig was close to the South-China-type miniature pig, whereas the Northern-China-type miniature pig belonged to one group and the plateau-type miniature pig belonged to another. The clustering patterns in the NJ tree reflect the geographical origins of the tested breeds; that is, breeds from neighboring regions had closer phylogenetic relationships with one another.

### Haplotype typing of swine leukocyte antigen microsatellites

In this study, the 18 microsatellites were divided according to the different SLA regions where they were located, and the haplotype frequency was calculated using the microsatellites in each SLA region.

**Figure 4:**
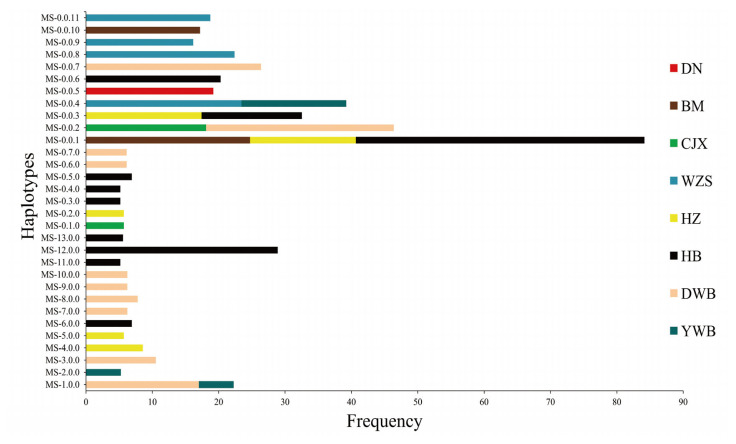
The haplotypes of microsatellites in different regions of the swine leukocyte antigen. The ordinate indicates the microsatellite haplotypes and the abscissa indicates frequency of the haplotypes. DN, Diannan small-ear pig; BM, Bama miniature pig; CJX, Congjiang Xiang pig; WZS, Wuzhishan pig; MG, Mingguang small-ear pig; HZ, Hezuo pig; HB, Hebao pig; DWB, Dongbei wild boar; YWB, Yunnan wild boar; HWB, Huanan wild boar.

A total of 480 non-zero haplotypes were obtained by haplotype analysis using six microsatellites in the SLA class I region, among which 13 common haplotypes (frequency >5%) were found (Figure 4; Supplementary Table S4). Among the common haplotypes, one haplotype (MS-1.0.0) was shared by Dongbei wild boar and Yunnan wild boar, and the other nine were specific haplotypes. It is worth noting that more than 5% of haplotype frequencies were obtained in only two Chinese miniature pig breeds (Hezuo and Hebao pigs) and two wild boar breeds (Dongbei and Yunnan wild boars).

A total of 640 non-zero haplotypes were obtained by haplotype analysis using nine microsatellites in the SLA class II region, among which seven common haplotypes (frequency >5%) were found (Figure 4; Supplementary Table S5). Interestingly, no shared haplotype was found in the seven common haplotypes, and all the common haplotypes were specific haplotypes. The results showed that more than 5% of haplotype frequencies were obtained in only three Chinese miniature pig breeds (Congjiang Xiang, Hezuo, and Hebao pigs) and one wild boar breed (Dongbei wild boar).

A total of 114 non-zero haplotypes were obtained by haplotype analysis using three microsatellites in the SLA class III region, among which 11 common haplotypes (frequency >15%) were found (Figure 4; Supplementary Table S6). The results showed that there were four shared haplotypes and seven haplotypes. It should be noted that the frequency of MS-0.0.1 was 43.50% in Hebao pigs, which indicated that this haplotype was the main haplotype in the SLA class III region of Hebao pigs.

Interestingly, no common haplotype was found in Ming guang small-ear pig and Huanan wild boar (haplotype frequency: SLA class I or SLA class II >5%, SLA class III >15%). In addition to common haplotypes, 467, 633, and 103 rare haplotypes were obtained in the SLA class I, II, and III regions, respectively (Supplementary file 1).

## DISCUSSION

### Microsatellite polymorphism in the swine leukocyte antigen regions

The highly variable polymorphisms of the SLA play an important role in swine anti-viral immune responses and affect the binding and presentation of peptide fragments to T lymphocytes [18]. The observed heterozygosity (Ho), He, and PIC can reflect population diversity. Ho is more susceptible to factors such as sample size, whereas He is less affected by sample size [19]. Therefore, the genetic polymorphism indices in this study were mainly evaluated by He and PIC. In this study, we found that almost all 18 microsatellite loci showed high polymorphism levels (except *SLAMS057* and *SLAMS094*), indicating that the immunity of Chinese indigenous pigs is strong. All SLA regions (I, II, and III) had medium-high polymorphism levels (Figure 2B), and the SLA I region had the highest polymorphism level, which is similar to the conclusion reported in a previous study [20]. The polymorphism level was lowest near the centromere region, which may be related to the presence of centromeres [20,21]. Our findings are consistent with the results of Smith et al [21], who showed that *SLAMS035*, *SLAMSA00*, and *SLAMS034* located near *SLA-1*, *2*, and *3* in SLA class I genes had high polymorphism levels. In this study, *SLAMS057* was located at the end of *SLA-6*, and the polymorphism level at this locus was low, which confirms that there were few polymorphisms in *SLA-6* [22].

The major histocompatibility complex, class II, DR Beta 1 (*DRB1*) and major histocompatibility complex, class II, DQ Beta 1 (*DQB1*) loci in the SLA II region showed very high polymorphism levels. Currently, the immune polymorphism database (IPD)-MHC (http://www.ebi.ac.uk/ipd/mhc/sla/) contains 99 *DRB1* alleles and 53 *DQB1* alleles. However, the *SLA*-major histocompatibility complex, class II, DQ Alpha (*DQA*) locus exhibits a moderate degree of polymorphism, and 26 alleles have been identified to date. However, the major histocompatibility complex, class II, DR Alpha (*DRA*) locus has low polymorphism levels and only 14 alleles. The *SLAMS052* and *SLAMS050* selected in this study were close to the *SLA-DRB* and *DQB* genes; therefore, both loci were highly polymorphic. *SLAMS051* was close to *SLA-DQA*, and this locus was moderately polymorphic. This conclusion is similar to that reported by Lunney et al [4], and it can also be speculated that these markers may have a certain linkage with these functional genes. In addition, although *SLAMS095* is closer to *SLA-DRA*, it is still a highly polymorphic locus, which may be due to the low degree of linkage between them.

### Intrapopulation genetic diversity

Many factors, such as the level of inbreeding, population size, the history or origin of the breeding population, the level of artificial selection pressure, and husbandry practices, affect the genetic diversity of domestic animal populations. The higher the genetic diversity or the richer the genetic variation, the stronger the ability to adapt to environmental changes; in contrast, breeds with low genetic diversity are more vulnerable to extinction [23]. In this study, we explored the genetic diversity of seven Chinese miniature pig breeds and three wild boar breeds based on 18 microsatellite loci in the SLA region. The high diversity of Chinese miniature pigs may be one of the most important reasons for their resistance to crude feed and strong disease resistance. However, the genetic diversity of Chinese miniature pigs was slightly lower than the wild boars, which is probably related to long-term breeding within the closed or small pig population.

It is worth noting that among the four pig types, the tran sitional type had the highest genetic diversity. From another point of view, the genetic diversity of Mingguang small-ear pigs was slightly lower than that of Diannan small-ear pigs. This phenomenon may be due to the inclusion of fewer transitional pig breeds and more South Chinese breeds in this study. The specific reasons for this need further exploration. In addition, the He (0.6834) of Diannan small-ear pigs in this study was similar to that found in a previous report by Fang et al [24] (He = 0.66) [24] but higher than that reported by Wang et al [25] (He = 0.5950). The He of Wuzhishan pigs in our study (0.6526) was similar to that reported by Wang et al [25] (He = 0.6446) but lower than that reported by Fang et al (He = 0.75).

### Interpopulation genetic diversity

Except for Mingguang small-ear pigs, the breeds in this study are clearly classified in “Animal Genetics Resources in China Pigs” [26]. The habitat altitude (from 1,000 m to 3,000 m) of the Mingguang small-ear pig is between those of the plateau-type and the South-China-type miniature pigs. Many phenotypic characteristics of Mingguang small-ear pigs are also between those of the two types; therefore, phenotypic characteristics are not suitable for classifying Mingguang small-ear pigs. Therefore, some scholars have classified Mingguang small-ear pigs as transition-type.

Fst measures the degree of differentiation between pop ulations. Among Chinese miniature pigs, the Fst value between Diannan small-ear pigs and Mingguang small-ear pigs was the lowest, indicating that there was almost no genetic difference between them. The Fst values of Hebao pigs compared with those of other breeds (including wild boars) were between 0.1379 and 0.2103. It is worth noting that both Hebao pigs and Dongbei wild boars were collected in Liaoning Province, but the Fst value between them (0.1686) was larger, which may be associated with selective breeding along with proper feeding over generations in that particular population. The DA and Dc distances are suitable for estimating the genetic relationships between populations based on microsatellite data [27,28]. In this study, we constructed an NJ tree using the DA and Dc distance methods to evaluate the genetic distance among different breeds. The South China-type miniature pigs were clustered into one group as a whole, which is consistent with previous reports [24,25]. The results showed that the genetic distance between Mingguang small-ear pigs and Diannan small-ear pigs was the smallest, suggesting that they have remarkably similar genetic backgrounds and originate from the same population. Hence, combined with the Fst results, we suggest that Mingguang small-ear pigs should be classified as South-China-type miniature pigs like Diannan small-ear pigs. However, our suggestions deviate from the traditional classification of breeds by some scholars. This may be because it is difficult to distinguish different breeds based solely on phenotypic traits.

In addition, Congjiang Xiang and Bama miniature pigs were clustered together, which is consistent with their geographical distribution. Although there was a great difference of Fst value between the Hebao pig and Dongbei wild boar, these two breeds were clustered together, which was also consistent with their geographical distribution. Overall, each breed in this study was clustered according to geographical distribution.

### Haplotype typing of swine leukocyte antigen microsatellites

Given the strong linkage disequilibrium exhibited by the SLA loci, it is sometimes more appropriate and convenient for researchers to communicate and present findings in terms of haplotypes rather than individual allele specificities [4]. To the best of our knowledge, at least 91 SLA class I haplotypes and 47 SLA class II haplotypes have been submitted to the ISAG SLA Nomenclature Committee [29]. A variety of molecular methods have been described for typing SLA alleles, including reverse transcription PCR (RT-PCR) sequence-based typing, PCR-sequence-specific primers (PCR-SSP), PCR-restriction fragment length polymorphism (PCR-RFLP), and MS. Many SLA class I and II haplotypes have been identified in different pig breeds and porcine cell lines using these methods [29–31]. Among them, the MS marker technique is a fast and cost-efficient method for molecular SLA typing [32]. Twenty-eight haplotypes in the SLA region were identified by 42 microsatellite loci from 72 individuals in six pig breeds, of which five haplotypes were confirmed by DNA sequencing in inbred NIH and Clawn miniature pigs [32]. Furthermore, 10 haplotypes in the SLA region, including three recombinant haplotypes, were observed at 22 microsatellite loci [31].

With reference to the nomenclature system established by the SLA Nomenclature Committee based on the high-resolution DNA sequencing method, we conducted preliminary naming of microsatellite haplotypes in the SLA region. SLA microsatellite haplotypes are named with the prefix “MS-,” and a number for the class I haplotype followed by two numbers for the class II and class III haplotypes, separated by a period (e.g., MS-1.1.1). The number “0” is assigned if there was no information on the associated class I and class II haplotypes (e.g., MS-0.0.1). In this study, we used 18 microsatellites for SLA typing, and no other common haplotypes were confirmed in previous studies. However, only one rare haplotype (MSHZ-0.0.15) of the Hezuo pig was confirmed in our study as a previous H04 haplotype [32]. This may be caused by breed differences, or it may be that the selected microsatellite loci are extremely polymorphic. It is worth noting that there were four, three, and three common haplotypes in each SLA region (I, II, and III) in Hebao pig (Figure 4), and their cumulative haplotype frequency exceeded 46.50%, 17.20%, and 78.90%, respectively. These results were in accordance with the slightly lower genetic diversity in the Hebao pig.

Overall, the SLA region had a relatively high genetic di versity in Chinese miniature pigs. Each breed was clustered according to geographical distribution. The Mingguang small-ear pig should belong to the same population as the Diannan small-ear pig. This study not only provided a reference for the protection of genetic resources and classification of Chinese miniature pigs but also provided information for the utilization of miniature pigs as an animal model.

## Figures and Tables

**Table 1:** Sample information in this study

Types	Population	Abbreviation	Number of samples
South China type	Diannan small-ear pig	DN	50
	Congjiang Xiang pig	CJX	35
	Bama miniature pig	BM	32
	Wuzhishan pig	WZS	32
Transitional type	Mingguang small-ear pig	MG	50
Plateau type	Hezuo pig	HZ	35
North China type	Hebao pig	HB	29
Wild boar	Yunnan wild boar	YWB	19
	Huanan wild boar	HWB	32
	Dongbei wild boar	DWB	32
	Total		346

**Table 2:** Comparison of genic diversity between Chinese miniature pigs and wild boars

Population	Type	Ho	He	PIC
Chinese miniature pigs	South China type	0.5149^a^	0.6434^a^	0.5963^a^
	Transitional type	0.5074^a^	0.6757^a^	0.6356^a^
	Plateau type	0.5363^a^	0.6387^a^	0.5930^a^
	North China type	0.4174^a^	0.4903^b^	0.4330^b^
	Mean	0.5029	0.6255	0.5781
Wild boar		0.5655	0.6530	0.6019

Ho, observed heterozygosity; He, expected heterozygosity; PIC, polymorphism information content.

a,b In the same column, means with different letter superscripts indicate significant difference (p<0.05).

**Table 3:** The comparison of Fst value among ten pig breeds

Population	MG	DN	CJX	BM	WZS	HZ	HB	YWB	HWB	DWB
MG	-									
DN	0.0299	-								
CJX	0.1055	0.1051	-							
BM	0.1022	0.1115	0.0630	-						
WZS	0.0707	0.0622	0.1220	0.1154	-					
HZ	0.1029	0.1007	0.0771	0.0610	0.1034	-				
HB	0.2088	0.2082	0.2065	0.1767	0.2074	0.1379	-			
YWB	0.0160	0.0215	0.1025	0.0921	0.0530	0.0902	0.2103	-		
HWB	0.0601	0.0455	0.1122	0.1200	0.0467	0.1015	0.2061	0.0396	-	
DWB	0.1152	0.1050	0.0924	0.0921	0.1096	0.0712	0.1686	0.0967	0.0974	-

DN, Diannan small-ear pig; CJX, Congjiang Xiang pig; BM, Bama miniature pig; WZS, Wuzhishan pig; MG, Mingguang small-ear pig; HZ, Hezuo pig; HB, Hebao pig; YWB, Yunnan wild boar; HWB, Huanan wild boar; DWB, Dongbei wild boar.
