# Supplementary material for: Genetic diversity analysis in Chinese miniature pigs using swine leukocyte antigen complex microsatellites
Source: Anim Biosci. 2021 Feb 16;34(11):1757–65. doi: 10.5713/ab.20.0637 (PMC8563246; doi:10.5713/ab.20.0637)
Supplement: Supplementary file 1 [file ab-20-0637-suppl1.pdf]

Appendix A. Supplementary data

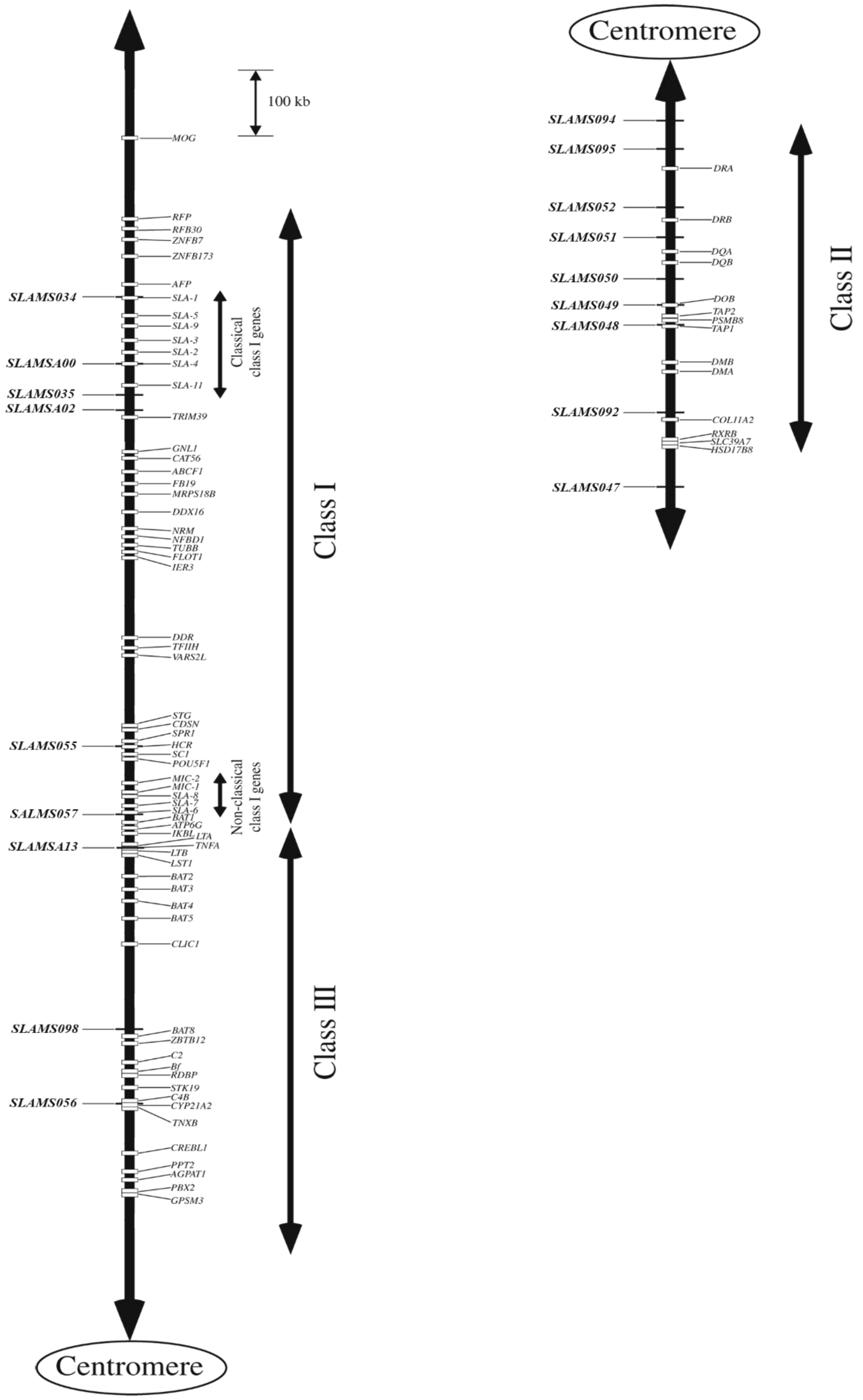

Supplementary Figure S1. Map of the locations of 18 MS markers (left of vertical arrow) and genes (right of vertical arrow) within the SLA genomic region.

Supplementary Table S1. The primer sequence of microsatellite locus

Name	Classes	Forward primer	Reverse primer	Repeat type	Annealing temp. (℃)
SLAMS047	Class II	(FAM) AAAAGAGGCAATGAGGTCCA	AGTGGGATGTTTTGGTCCAG	$(CA)_{14}$	56
SLAMS092	Class II	(FAM) TCTCTCTCCCTCCCACTCTG	CTGCTCTCATTGCCTTCCTT	$(CA)_{21}$	56
SLAMS048	Class II	(FAM) CCTTCACCTTCCCAGAACCT	CTCCCAGCCTCACCTACCTC	$(TG)_{14}$	56
SLAMS049	Class II	(FAM) AATAGAAGCGCCTCCAAAGA	CAAGGTGATGGGAAATACAAGG	$(GA)_{27}$	56
SLAMS050	Class II	(FAM) CTCTCGGTCCCATAACTCTGA	TGAGAGGGGAGACAACAACC	$(TA)_{24}$	59.8
SLAMS051	Class II	(FAM) TGCTATGTTTCCTTGTCATAATCT	TGTTCAGCTCAATGATTTGGAG	$(TTTA)_{6}$	58.4
SLAMS052	Class II	(FAM) TGTGCAGACTAGGAGTGAGGA	CACAGGGACACCAAGGATG	$(TG)_{13}$	56
SLAMS095	Class II	(FAM) TCATGGCTGTGACCTTGGTA	CCTGCTCCTTGCTCTGTAGG	$(TG)_{13}TA(TG)_{5}$	56
SLAMS094	Class II	(FAM) TCCAACTGTCTGGGACTCCT	TAGTGGGGGAAATGGAATTG	$(TTTATTTG)_{2}(TTTA)_{3}$	56
SLAMS056	Class III	(FAM) CAACCACGAAGACCTGCCTAAT	GTTGCTGTAGCTCTGGTGTAGA	$(CA)_{15}$	56
SLAMS098	Class III	(FAM) GACAGGCAGTCACCAGAACA	CTCCACCCACCTATTGCCTA	$(TTG)_{5}$	56

SLAMSA13	Class III	(FAM) AACAACCCAGCAAGGAGATG	TGAGGCCAAGATAGAGACCAA	$(CAG)_{22}$	56
SLAMS057	Class I	(FAM) AAGCTGTGATTCCTAACCTG	GGGGTACCACATAGAGATTC	$(TAA)_6$	56
SLAMS055	Class I	(FAM) CACTCAGATCTGACGTTGCTGT	CATGGTTAAGAAACTTCCATA	$(AAAT)_6$	56
SLAMSA02	Class I	(FAM) CAGGAAGTCATATTTGGCAAGA	CATGTAGCATTAGTGTCCCTGGT	$(TTG)_5$	56
SLAMS035	Class I	(FAM) CCTGTGTTTCTATGGCTGTGC	CAGGGAAGGAACCCACATC	$(CA)_{20}$	56
SLAMSA00	Class I	(FAM) GTGGTTTCTGGGCAGATGA	TTGGACATGATCTGTCAGGTG	$(TG)_{10}$	56
SLAMS034	Class I	(FAM) CCAGGTGGCTGACTTAGGAG	TCCTTTTGCCCACAAAGAAC	$(CA)_9$	56

Supplementary Table S2. Comparison of genetic diversity between different breeds of miniature pigs and wild boars

Parameters	DN	CJX	BM	WZS	MG	HZ	HB	YWB	HWB	DWB
Ho	0.5241	0.4689	0.4966	0.5700	0.5074	0.5363	0.4174	0.5308	0.5619	0.6037
He	0.6834	0.5948	0.6429	0.6526	0.6757	0.6387	0.4903	0.6737	0.6654	0.6200
PIC	0.6396	0.5476	0.5947	0.6033	0.6356	0.5930	0.4330	0.6219	0.6192	0.5646
DHWE	13	12	8	6	13	9	8	8	10	3

Abbreviations: Ho, observed heterozygosity; He, expected heterozygosity; PIC, polymorphism information content; DHWE, of loci deviating from Hardy–Weinberg equilibrium (DHWE) at $P < 0.05$ among ten populations. The population breed abbreviations are defined in Table 1, the same as below.

Supplementary Table S3. The genetic distance among ten pig breeds

Population	MG	DN	CJX	BM	WZS	HZ	HB	YWB	HWB	DWB
MG	-	0.0873	0.2104	0.2068	0.1811	0.2197	0.3412	0.1160	0.1721	0.2317
DN	0.2541	-	0.1987	0.1907	0.1786	0.1959	0.3141	0.1007	0.1570	0.2106
CJX	0.3889	0.3794	-	0.1603	0.2182	0.2280	0.3042	0.2244	0.2150	0.2300
BM	0.3787	0.3662	0.3321	-	0.2063	0.1431	0.2625	0.1861	0.2270	0.1958
WZS	0.3534	0.3569	0.3818	0.3812	-	0.2252	0.3053	0.1935	0.1397	0.1752
HZ	0.3918	0.3633	0.3920	0.3107	0.3965	-	0.2162	0.2062	0.2288	0.1880
HB	0.4972	0.4781	0.4468	0.4198	0.4628	0.3883	-	0.3199	0.3034	0.2126
YWB	0.2814	0.2700	0.4032	0.3550	0.3660	0.3913	0.4859	-	0.1805	0.2258
HWB	0.3495	0.3417	0.3907	0.4096	0.3019	0.4095	0.4660	0.3664	-	0.1670
DWB	0.4116	0.3802	0.3863	0.3697	0.3390	0.3596	0.3741	0.4106	0.3346	-

Note: Below diagonal: Dc distance, Above diagonal: DA distance.

Supplementary Table S4. Haplotypes of SLA Class I based on microsatellite typing (frequency > 5%)

Haplotypes	Population	Frequencies (%)	Name of MS marker: SLAMS					
			057	055	A02	035	A00	034
MS-1.0.0	DWB	17.01	117	220	403	230	213	307
	YWB	5.26						
MS-2.0.0	YWB	5.26	117	216	397	236	211	309
MS-3.0.0	YWB	10.53	117	216	400	232	215	309
MS-4.0.0	HZ	8.57	117	216	391	242	213	307
MS-5.0.0	HZ	5.71	117	216	400	234	213	307
MS-6.0.0	HB	6.90	117	220	403	238	213	307
MS-7.0.0	DWB	6.25	117	212	400	230	215	309
MS-8.0.0	DWB	7.81	117	220	400	228	215	307
MS-9.0.0	DWB	6.25	117	220	400	232	215	309
MS-10.0.0	DWB	6.25	117	220	403	236	213	309
MS-11.0.0	HB	5.17	111	216	403	210	215	307
MS-12.0.0	HB	28.90	111	220	403	210	215	307
MS-13.0.0	HB	5.59	111	220	403	210	215	309

Supplementary Table S5. Haplotypes of SLA Class II based on microsatellite typing (frequency > 5%)

Haplotypes	Population	Frequencies (%)	Name of MS marker: SLAMS								
			047	092	048	049	050	051	052	095	094
MS-0.1.0	CJX	5.71	235	298	197	295	143	262	232	230	391
MS-0.2.0	HZ	5.71	241	288	197	295	125	262	234	232	391
MS-0.3.0	HB	5.17	215	298	201	297	119	262	236	228	391
MS-0.4.0	HB	5.17	215	298	201	297	119	266	232	228	391
MS-0.5.0	HB	6.90	241	296	185	287	119	266	220	228	391
MS-0.6.0	DWB	6.14	211	294	195	293	125	266	220	232	391
MS-0.7.0	DWB	6.14	241	292	185	287	131	274	226	232	391

Supplementary Table S6. Haplotypes of SLA Class III based on microsatellite typing (frequency > 15%)

Haplotypes	Population	Frequencies (%)	Name of MS marker: SLAMS		
			056	098	A13
MS-0.0.1	HZ	15.93	276	335	203
	BM	24.73			
	HB	43.50			
MS-0.0.2	CJX	18.11	276	338	203
	DWB	28.29			
MS-0.0.3	HZ	17.42	276	335	200
	HB	15.12			
MS-0.0.4	YWB	15.79	274	335	200
	WZS	23.44			
MS-0.0.5	DN	19.20	274	338	203
MS-0.0.6	HB	20.29	276	332	203
MS-0.0.7	LNY	26.40	276	338	200
MS-0.0.8	CJX	22.40	276	335	209
MS-0.0.9	CJX	16.17	276	338	209
MS-0.0.10	BM	17.19	276	338	230
MS-0.0.11	WZS	18.75	274	338	200
