# Supplementary material for: Genetic diversity analysis in Chinese miniature pigs using swine leukocyte antigen complex microsatellites
Source: Anim Biosci. 2021 Feb 16;34(11):1757–65. doi: 10.5713/ab.20.0637 (PMC8563246; doi:10.5713/ab.20.0637)
Supplement: Supplementary file 2 [file ab-20-0637-suppl2.pdf]

#1 Rare haplotypes of SLA Class I region based on microsatellite typing (Frequency < 5%, ND: Not detected)

Population	Rare Haplotypes	Frequencies	Name of MS marker: SLAMS					
			057	055	A02	035	A00	034
Diannan small-ear pig	MSDN-1.0.0	0.0100	111	212	403	226	213	307
	MSDN-2.0.0	0.0100	111	216	397	226	211	307
	MSDN-3.0.0	0.0100	111	216	400	224	211	307
	MSDN-4.0.0	0.0100	111	216	400	226	213	311
	MSDN-5.0.0	0.0100	111	216	400	228	215	307
	MSDN-6.0.0	0.0100	111	216	400	240	209	309
	MSDN-7.0.0	0.0100	111	216	400	244	213	307
	MSDN-8.0.0	0.0200	111	216	403	224	211	309
	MSDN-9.0.0	0.0100	111	216	403	238	211	309
	MSDN-10.0.0	0.0100	111	220	400	224	213	307
	MSDN-11.0.0	0.0100	111	220	400	224	213	309
	MSDN-12.0.0	0.0100	111	220	400	232	213	315
	MSDN-13.0.0	0.0100	111	220	400	242	213	309
	MSDN-14.0.0	0.0200	111	220	403	224	211	309
	MSDN-15.0.0	0.0100	111	220	403	240	213	309
	MSDN-16.0.0	0.0100	111	220	403	250	215	309
	MSDN-17.0.0	0.0100	111	228	400	226	213	319
	MSDN-18.0.0	0.0100	111	228	403	230	213	307
	MSDN-19.0.0	0.0100	111	ND	397	230	211	ND
	MSDN-20.0.0	0.0100	111	ND	397	230	211	ND
	MSDN-21.0.0	0.0100	111	ND	403	242	209	309
	MSDN-22.0.0	0.0100	117	204	400	240	209	309
	MSDN-23.0.0	0.0100	117	212	400	208	213	315
	MSDN-24.0.0	0.0100	117	212	400	232	213	309
	MSDN-25.0.0	0.0100	117	212	400	234	211	315
	MSDN-26.0.0	0.0100	117	212	403	224	211	307
	MSDN-27.0.0	0.0100	117	212	403	230	213	311
	MSDN-28.0.0	0.0100	117	212	403	232	217	309
	MSDN-29.0.0	0.0100	117	212	403	246	213	307
	MSDN-30.0.0	0.0100	117	212	403	250	215	311
	MSDN-31.0.0	0.0100	117	216	397	230	213	307
	MSDN-32.0.0	0.0100	117	216	400	208	213	311
	MSDN-33.0.0	0.0100	117	216	400	212	213	319
	MSDN-34.0.0	0.0100	117	216	400	220	209	315
	MSDN-35.0.0	0.0200	117	216	400	224	207	309
	MSDN-36.0.0	0.0100	117	216	400	224	211	309
	MSDN-37.0.0	0.0114	117	216	400	224	213	307
	MSDN-38.0.0	0.0286	117	216	400	224	213	309
	MSDN-39.0.0	0.0100	117	216	400	226	215	309
	MSDN-40.0.0	0.0100	117	216	400	228	207	307
	MSDN-41.0.0	0.0100	117	216	400	228	207	309
	MSDN-42.0.0	0.0100	117	216	400	228	215	307
	MSDN-43.0.0	0.0286	117	216	400	230	213	307
	MSDN-44.0.0	0.0114	117	216	400	230	213	309
	MSDN-45.0.0	0.0200	117	216	400	230	215	309
	MSDN-46.0.0	0.0200	117	216	400	232	217	309
	MSDN-47.0.0	0.0100	117	216	400	242	209	307
	MSDN-48.0.0	0.0100	117	216	400	242	211	311
	MSDN-49.0.0	0.0100	117	216	400	244	213	309
	MSDN-50.0.0	0.0100	117	216	400	246	215	309
	MSDN-51.0.0	0.0300	117	216	403	208	215	307
	MSDN-52.0.0	0.0100	117	216	403	222	211	311
	MSDN-53.0.0	0.0100	117	216	403	224	213	311
	MSDN-54.0.0	0.0200	117	216	403	226	213	311

	MSDN-55.0.0	0.0100	117	216	403	228	213	309
	MSDN-56.0.0	0.0300	117	216	403	228	213	311
	MSDN-57.0.0	0.0100	117	216	403	232	213	307
	MSDN-58.0.0	0.0100	117	216	403	234	219	309
	MSDN-59.0.0	0.0100	117	216	403	240	215	307
	MSDN-60.0.0	0.0100	117	216	403	242	215	309
	MSDN-61.0.0	0.0200	117	220	397	228	213	311
	MSDN-62.0.0	0.0100	117	220	397	230	211	311
	MSDN-63.0.0	0.0100	117	220	400	208	217	307
	MSDN-64.0.0	0.0100	117	220	400	216	213	309
	MSDN-65.0.0	0.0100	117	220	400	224	215	307
	MSDN-66.0.0	0.0200	117	220	400	224	215	309
	MSDN-67.0.0	0.0100	117	220	400	226	213	315
	MSDN-68.0.0	0.0100	117	220	400	226	215	307
	MSDN-69.0.0	0.0100	117	220	400	228	215	307
	MSDN-70.0.0	0.0100	117	220	400	234	211	311
	MSDN-71.0.0	0.0100	117	220	400	238	213	307
	MSDN-72.0.0	0.0100	117	220	400	238	213	311
	MSDN-73.0.0	0.0100	117	220	403	224	213	315
	MSDN-74.0.0	0.0100	117	220	403	224	215	309
	MSDN-75.0.0	0.0100	117	220	403	226	211	307
	MSDN-76.0.0	0.0100	117	220	403	230	209	307
	MSDN-77.0.0	0.0100	117	220	403	230	211	309
	MSDN-78.0.0	0.0100	117	220	403	238	215	307
	MSDN-79.0.0	0.0100	117	220	403	246	211	311
	MSDN-80.0.0	0.0100	117	220	403	250	215	311
	MSDN-81.0.0	0.0100	117	228	400	220	213	309
	MSDN-82.0.0	0.0100	117	ND	400	244	211	309
	MSDN-83.0.0	0.0100	ND	220	400	208	215	307
	MSDN-84.0.0	0.0100	ND	220	400	224	215	317
Congjiang Xiang pig	MSCJX-1.0.0	0.0143	111	208	400	214	213	307
	MSCJX-2.0.0	0.0143	111	216	400	230	213	305
	MSCJX-3.0.0	0.0143	111	220	400	214	213	307
	MSCJX-4.0.0	0.0143	111	220	400	226	215	309
	MSCJX-5.0.0	0.0143	111	220	400	226	217	307
	MSCJX-6.0.0	0.0143	111	220	403	236	213	307
	MSCJX-7.0.0	0.0143	111	220	403	242	213	307
	MSCJX-8.0.0	0.0143	111	ND	391	ND	213	309
	MSCJX-9.0.0	0.0143	111	ND	400	220	215	309
	MSCJX-10.0.0	0.0143	111	ND	400	242	215	309
	MSCJX-11.0.0	0.0143	111	ND	403	228	215	307
	MSCJX-12.0.0	0.0143	111	ND	403	ND	217	309
	MSCJX-13.0.0	0.0143	117	200	391	214	213	309
	MSCJX-14.0.0	0.0143	117	216	400	220	217	307
	MSCJX-15.0.0	0.0143	117	216	400	228	215	311
	MSCJX-16.0.0	0.0286	117	216	400	230	215	307
	MSCJX-17.0.0	0.0143	117	216	400	232	213	309
	MSCJX-18.0.0	0.0143	117	220	391	232	215	307
	MSCJX-19.0.0	0.0143	117	220	400	214	213	307
	MSCJX-20.0.0	0.0143	117	220	400	214	213	311
	MSCJX-21.0.0	0.0143	117	220	400	214	215	307
	MSCJX-22.0.0	0.0286	117	220	400	214	215	309
	MSCJX-23.0.0	0.0286	117	220	400	220	213	307
	MSCJX-24.0.0	0.0143	117	220	400	220	213	309
	MSCJX-25.0.0	0.0143	117	220	400	220	215	307
	MSCJX-26.0.0	0.0143	117	220	400	222	213	307
	MSCJX-27.0.0	0.0143	117	220	400	224	215	309
	MSCJX-28.0.0	0.0429	117	220	400	226	213	307

	MSCJX-29.0.0	0.0286	117	220	400	226	215	309
	MSCJX-30.0.0	0.0143	117	220	400	226	215	315
	MSCJX-31.0.0	0.0286	117	220	400	228	213	309
	MSCJX-32.0.0	0.0286	117	220	400	230	215	309
	MSCJX-33.0.0	0.0143	117	220	400	230	217	309
	MSCJX-34.0.0	0.0143	117	220	400	236	215	307
	MSCJX-35.0.0	0.0143	117	220	400	246	213	309
	MSCJX-36.0.0	0.0143	117	220	403	220	213	307
	MSCJX-37.0.0	0.0143	117	220	403	226	217	307
	MSCJX-38.0.0	0.0143	117	220	403	240	215	307
	MSCJX-39.0.0	0.0143	117	220	406	220	217	309
	MSCJX-40.0.0	0.0143	117	220	406	230	213	319
	MSCJX-41.0.0	0.0143	117	224	400	210	215	305
	MSCJX-42.0.0	0.0143	117	224	400	228	217	307
	MSCJX-43.0.0	0.0143	117	228	400	226	215	307
Bama miniature pig	MSBM-1.0.0	0.0156	111	212	400	228	215	309
	MSBM-2.0.0	0.0156	111	212	400	232	215	315
	MSBM-3.0.0	0.0156	111	216	391	232	213	315
	MSBM-4.0.0	0.0156	111	220	391	228	213	307
	MSBM-5.0.0	0.0156	111	ND	397	230	217	307
	MSBM-6.0.0	0.0156	114	212	400	230	215	307
	MSBM-7.0.0	0.0156	114	212	403	ND	209	309
	MSBM-8.0.0	0.0156	114	216	397	230	213	309
	MSBM-9.0.0	0.0156	114	216	400	230	213	307
	MSBM-10.0.0	0.0156	114	216	400	232	215	309
	MSBM-11.0.0	0.0313	114	216	403	232	215	307
	MSBM-12.0.0	0.0156	114	216	406	228	213	309
	MSBM-13.0.0	0.0156	114	216	406	234	215	305
	MSBM-14.0.0	0.0469	114	220	403	210	215	315
	MSBM-15.0.0	0.0156	114	220	403	242	215	307
	MSBM-16.0.0	0.0156	114	ND	397	230	213	307
	MSBM-17.0.0	0.0156	114	ND	400	228	215	309
	MSBM-18.0.0	0.0313	117	212	397	228	213	309
	MSBM-19.0.0	0.0156	117	212	400	230	213	309
	MSBM-20.0.0	0.0156	117	212	400	232	215	307
	MSBM-21.0.0	0.0156	117	212	403	246	213	307
	MSBM-22.0.0	0.0156	117	212	403	ND	213	307
	MSBM-23.0.0	0.0156	117	216	391	208	215	307
	MSBM-24.0.0	0.0313	117	216	391	230	213	307
	MSBM-25.0.0	0.0469	117	216	397	230	213	309
	MSBM-26.0.0	0.0156	117	216	397	232	213	307
	MSBM-27.0.0	0.0469	117	216	400	228	213	307
	MSBM-28.0.0	0.0156	117	216	400	228	215	309
	MSBM-29.0.0	0.0469	117	216	400	232	215	307
	MSBM-30.0.0	0.0156	117	216	400	234	213	309
	MSBM-31.0.0	0.0156	117	216	403	210	215	315
	MSBM-32.0.0	0.0156	117	216	403	228	227	305
	MSBM-33.0.0	0.0156	117	216	403	234	213	307
	MSBM-34.0.0	0.0156	117	216	403	238	213	309
	MSBM-35.0.0	0.0156	117	216	406	228	209	305
	MSBM-36.0.0	0.0156	117	220	391	244	213	307
	MSBM-37.0.0	0.0156	117	220	397	228	213	309
	MSBM-38.0.0	0.0156	117	220	400	228	213	307
	MSBM-39.0.0	0.0313	117	220	400	228	215	307
	MSBM-40.0.0	0.0156	117	220	400	230	213	309
	MSBM-41.0.0	0.0156	117	220	400	246	215	309
	MSBM-42.0.0	0.0313	117	220	403	232	215	307
	MSBM-43.0.0	0.0156	117	220	403	244	213	315

	MSBM-44.0.0	0.0156	117	220	403	246	213	307
	MSBM-45.0.0	0.0156	117	ND	391	246	215	307
	MSBM-46.0.0	0.0156	117	ND	400	232	213	307
	MSBM-47.0.0	0.0313	117	ND	403	220	215	315
	MSBM-48.0.0	0.0156	117	ND	403	252	217	309
	MSBM-49.0.0	0.0156	ND	220	391	ND	211	315
	MSBM-50.0.0	0.0156	ND	220	400	ND	211	315
Wuzhishan pig	MSWZS-1.0.0	0.0156	111	212	400	236	215	307
	MSWZS-2.0.0	0.0156	111	212	403	230	211	303
	MSWZS-3.0.0	0.0156	111	216	403	240	213	309
	MSWZS-4.0.0	0.0156	111	220	400	228	215	307
	MSWZS-5.0.0	0.0156	111	220	400	244	211	307
	MSWZS-6.0.0	0.0156	111	220	403	236	213	319
	MSWZS-7.0.0	0.0156	111	220	403	240	213	309
	MSWZS-8.0.0	0.0156	111	220	403	242	219	319
	MSWZS-9.0.0	0.0313	111	220	403	246	213	307
	MSWZS-10.0.0	0.0156	111	220	ND	242	211	307
	MSWZS-11.0.0	0.0156	111	220	ND	242	ND	307
	MSWZS-12.0.0	0.0156	111	224	403	232	211	307
	MSWZS-13.0.0	0.0156	111	224	403	232	213	305
	MSWZS-14.0.0	0.0156	111	224	403	242	215	307
	MSWZS-15.0.0	0.0156	111	224	406	234	215	307
	MSWZS-16.0.0	0.0156	111	ND	403	232	213	307
	MSWZS-17.0.0	0.0156	114	212	400	ND	215	303
	MSWZS-18.0.0	0.0156	117	212	400	ND	ND	319
	MSWZS-19.0.0	0.0469	117	212	403	230	213	307
	MSWZS-20.0.0	0.0156	117	212	403	232	215	307
	MSWZS-21.0.0	0.0156	117	212	ND	224	213	309
	MSWZS-22.0.0	0.0156	117	216	400	224	217	309
	MSWZS-23.0.0	0.0156	117	216	400	230	213	307
	MSWZS-24.0.0	0.0156	117	216	403	230	213	307
	MSWZS-25.0.0	0.0313	117	220	400	224	213	307
	MSWZS-26.0.0	0.0313	117	220	400	226	215	307
	MSWZS-27.0.0	0.0156	117	220	400	234	217	307
	MSWZS-28.0.0	0.0156	117	220	400	246	217	307
	MSWZS-29.0.0	0.0156	117	220	400	ND	211	309
	MSWZS-30.0.0	0.0156	117	220	400	ND	ND	307
	MSWZS-31.0.0	0.0313	117	220	403	234	213	307
	MSWZS-32.0.0	0.0156	117	220	403	238	213	319
	MSWZS-33.0.0	0.0313	117	220	403	242	211	307
	MSWZS-34.0.0	0.0469	117	220	403	242	213	307
	MSWZS-35.0.0	0.0156	117	220	403	246	211	309
	MSWZS-36.0.0	0.0156	117	220	406	232	213	307
	MSWZS-37.0.0	0.0313	117	220	406	232	215	307
	MSWZS-38.0.0	0.0156	117	220	ND	224	213	309
	MSWZS-39.0.0	0.0156	117	220	ND	234	ND	319
	MSWZS-40.0.0	0.0156	117	220	ND	244	217	307
	MSWZS-41.0.0	0.0156	117	224	400	224	215	307
	MSWZS-42.0.0	0.0156	117	224	400	230	213	307
	MSWZS-43.0.0	0.0313	117	224	400	232	217	307
	MSWZS-44.0.0	0.0156	117	224	403	236	217	307
	MSWZS-45.0.0	0.0156	117	ND	391	228	217	309
	MSWZS-46.0.0	0.0156	117	ND	400	236	215	309
	MSWZS-47.0.0	0.0313	117	ND	400	242	213	307
	MSWZS-48.0.0	0.0156	117	ND	403	230	219	307
	MSWZS-49.0.0	0.0156	117	ND	403	242	213	319
	MSWZS-50.0.0	0.0156	117	ND	406	228	215	307
	MSWZS-51.0.0	0.0156	117	ND	ND	224	213	307

	MSWZS-52.0.0	0.0156	117	ND	ND	230	213	307
Mingguang small-ear pig	MSMG-1.0.0	0.0100	111	212	400	226	219	309
	MSMG-2.0.0	0.0100	111	212	403	244	213	309
	MSMG-3.0.0	0.0100	111	216	400	232	213	309
	MSMG-4.0.0	0.0100	111	216	400	236	213	309
	MSMG-5.0.0	0.0100	111	220	397	214	211	307
	MSMG-6.0.0	0.0100	111	220	397	226	215	309
	MSMG-7.0.0	0.0100	111	220	397	226	217	307
	MSMG-8.0.0	0.0100	111	220	400	224	211	309
	MSMG-9.0.0	0.0100	111	220	400	230	209	307
	MSMG-10.0.0	0.0100	111	220	400	236	213	307
	MSMG-11.0.0	0.0100	111	220	403	212	211	309
	MSMG-12.0.0	0.0200	111	220	403	230	209	309
	MSMG-13.0.0	0.0200	111	220	403	230	211	307
	MSMG-14.0.0	0.0100	111	220	ND	230	219	309
	MSMG-15.0.0	0.0100	111	224	400	230	213	305
	MSMG-16.0.0	0.0100	114	216	400	208	217	309
	MSMG-17.0.0	0.0100	114	216	403	238	213	309
	MSMG-18.0.0	0.0100	114	220	400	232	211	307
	MSMG-19.0.0	0.0100	114	220	400	234	211	307
	MSMG-20.0.0	0.0100	117	212	391	238	213	309
	MSMG-21.0.0	0.0100	117	212	400	228	207	307
	MSMG-22.0.0	0.0100	117	216	391	238	211	307
	MSMG-23.0.0	0.0200	117	216	397	226	219	309
	MSMG-24.0.0	0.0100	117	216	397	234	211	309
	MSMG-25.0.0	0.0100	117	216	400	230	211	305
	MSMG-26.0.0	0.0300	117	216	400	230	211	309
	MSMG-27.0.0	0.0100	117	216	400	236	209	309
	MSMG-28.0.0	0.0100	117	216	403	208	213	309
	MSMG-29.0.0	0.0100	117	216	403	216	219	307
	MSMG-30.0.0	0.0100	117	220	397	214	227	307
	MSMG-31.0.0	0.0100	117	220	397	216	211	309
	MSMG-32.0.0	0.0100	117	220	397	224	213	307
	MSMG-33.0.0	0.0100	117	220	397	226	211	307
	MSMG-34.0.0	0.0300	117	220	397	226	211	309
	MSMG-35.0.0	0.0100	117	220	397	226	215	309
	MSMG-36.0.0	0.0100	117	220	397	226	227	309
	MSMG-37.0.0	0.0124	117	220	397	228	213	309
	MSMG-38.0.0	0.0200	117	220	397	230	211	309
	MSMG-39.0.0	0.0200	117	220	397	232	211	309
	MSMG-40.0.0	0.0176	117	220	397	232	213	309
	MSMG-41.0.0	0.0100	117	220	397	234	209	307
	MSMG-42.0.0	0.0100	117	220	397	234	211	309
	MSMG-43.0.0	0.0100	117	220	397	242	211	311
	MSMG-44.0.0	0.0100	117	220	400	214	211	307
	MSMG-45.0.0	0.0300	117	220	400	214	213	309
	MSMG-46.0.0	0.0200	117	220	400	218	219	307
	MSMG-47.0.0	0.0100	117	220	400	220	211	309
	MSMG-48.0.0	0.0100	117	220	400	224	211	317
	MSMG-49.0.0	0.0400	117	220	400	224	213	309
	MSMG-50.0.0	0.0100	117	220	400	226	211	309
	MSMG-51.0.0	0.0276	117	220	400	228	211	309
	MSMG-52.0.0	0.0100	117	220	400	230	209	309
	MSMG-53.0.0	0.0300	117	220	400	230	211	307
	MSMG-54.0.0	0.0100	117	220	400	230	213	309
	MSMG-55.0.0	0.0100	117	220	400	232	209	307
	MSMG-56.0.0	0.0100	117	220	400	232	209	309
	MSMG-57.0.0	0.0124	117	220	400	232	211	309

	MSMG-58.0.0	0.0100	117	220	400	234	213	307
	MSMG-59.0.0	0.0100	117	220	400	242	211	311
	MSMG-60.0.0	0.0100	117	220	400	242	213	309
	MSMG-61.0.0	0.0100	117	220	403	212	215	307
	MSMG-62.0.0	0.0100	117	220	403	214	209	309
	MSMG-63.0.0	0.0100	117	220	403	214	209	317
	MSMG-64.0.0	0.0100	117	220	403	226	209	317
	MSMG-65.0.0	0.0100	117	220	403	232	211	309
	MSMG-66.0.0	0.0200	117	220	403	234	213	309
	MSMG-67.0.0	0.0100	117	220	403	234	217	309
	MSMG-68.0.0	0.0100	117	220	403	242	217	309
	MSMG-69.0.0	0.0200	117	220	403	242	219	307
	MSMG-70.0.0	0.0100	117	220	403	246	209	309
	MSMG-71.0.0	0.0100	117	220	406	240	211	309
	MSMG-72.0.0	0.0100	117	220	ND	230	217	309
	MSMG-73.0.0	0.0100	117	224	400	216	213	307
	MSMG-74.0.0	0.0100	117	224	403	222	215	309
	MSMG-75.0.0	0.0100	ND	216	403	216	209	311
	MSMG-76.0.0	0.0100	ND	220	391	210	209	311
	MSMG-77.0.0	0.0100	ND	220	391	212	209	311
	MSMG-78.0.0	0.0100	ND	220	400	218	209	309
Hezuo pig	MSHZ-1.0.0	0.0143	111	212	400	234	215	303
	MSHZ-2.0.0	0.0143	111	216	400	210	213	307
	MSHZ-3.0.0	0.0143	111	216	400	234	213	307
	MSHZ-4.0.0	0.0286	111	216	400	236	215	307
	MSHZ-5.0.0	0.0143	111	216	400	248	213	319
	MSHZ-6.0.0	0.0143	111	216	403	230	213	307
	MSHZ-7.0.0	0.0143	111	216	403	230	ND	309
	MSHZ-8.0.0	0.0143	111	ND	400	230	215	309
	MSHZ-9.0.0	0.0143	114	212	400	246	215	309
	MSHZ-10.0.0	0.0143	114	212	403	212	215	307
	MSHZ-11.0.0	0.0143	114	212	403	236	213	309
	MSHZ-12.0.0	0.0143	114	216	391	242	213	307
	MSHZ-13.0.0	0.0143	114	216	400	228	213	319
	MSHZ-14.0.0	0.0143	114	216	400	230	215	307
	MSHZ-15.0.0	0.0143	114	216	400	232	215	309
	MSHZ-16.0.0	0.0143	114	216	403	218	213	307
	MSHZ-17.0.0	0.0143	114	216	403	240	215	307
	MSHZ-18.0.0	0.0143	117	212	400	212	211	307
	MSHZ-19.0.0	0.0143	117	212	400	230	215	307
	MSHZ-20.0.0	0.0143	117	212	400	230	215	309
	MSHZ-21.0.0	0.0143	117	212	403	218	211	309
	MSHZ-22.0.0	0.0143	117	212	403	242	213	307
	MSHZ-23.0.0	0.0143	117	216	391	230	213	307
	MSHZ-24.0.0	0.0143	117	216	400	210	213	307
	MSHZ-25.0.0	0.0143	117	216	400	228	215	307
	MSHZ-26.0.0	0.0143	117	216	400	232	213	309
	MSHZ-27.0.0	0.0286	117	216	400	232	215	319
	MSHZ-28.0.0	0.0143	117	216	400	234	215	303
	MSHZ-29.0.0	0.0143	117	216	400	234	ND	309
	MSHZ-30.0.0	0.0286	117	216	400	244	215	307
	MSHZ-31.0.0	0.0143	117	216	400	244	ND	309
	MSHZ-32.0.0	0.0143	117	216	403	218	213	307
	MSHZ-33.0.0	0.0143	117	216	403	230	ND	307
	MSHZ-34.0.0	0.0143	117	216	403	238	215	319
	MSHZ-35.0.0	0.0143	117	216	403	238	ND	307
	MSHZ-36.0.0	0.0143	117	216	403	238	ND	309
	MSHZ-37.0.0	0.0143	117	216	403	244	215	309

	MSHZ-38.0.0	0.0143	117	216	403	244	ND	311
	MSHZ-39.0.0	0.0143	117	216	403	246	211	309
	MSHZ-40.0.0	0.0143	117	216	403	246	215	307
	MSHZ-41.0.0	0.0429	117	216	406	232	213	307
	MSHZ-42.0.0	0.0286	117	216	406	234	213	307
	MSHZ-43.0.0	0.0143	117	216	406	236	ND	319
	MSHZ-44.0.0	0.0143	117	220	400	210	213	309
	MSHZ-45.0.0	0.0143	117	220	400	210	215	309
	MSHZ-46.0.0	0.0143	117	220	403	218	213	309
	MSHZ-47.0.0	0.0143	117	220	403	238	213	307
	MSHZ-48.0.0	0.0286	117	ND	391	242	213	307
	MSHZ-49.0.0	0.0286	117	ND	400	234	213	307
	MSHZ-50.0.0	0.0143	117	ND	400	236	213	309
	MSHZ-51.0.0	0.0286	117	ND	400	236	215	307
Hebao pig	MSHB-1.0.0	0.0172	105	220	400	208	217	307
	MSHB-2.0.0	0.0172	105	220	403	210	215	307
	MSHB-3.0.0	0.0172	105	220	403	210	215	309
	MSHB-4.0.0	0.0345	108	220	403	210	215	307
	MSHB-5.0.0	0.0172	111	212	403	228	215	307
	MSHB-6.0.0	0.0172	111	212	403	236	215	307
	MSHB-7.0.0	0.0172	111	220	403	204	ND	307
	MSHB-8.0.0	0.0172	111	220	403	208	227	307
	MSHB-9.0.0	0.0345	111	220	403	210	ND	307
	MSHB-10.0.0	0.0345	111	220	403	210	ND	309
	MSHB-11.0.0	0.0172	114	212	400	234	213	309
	MSHB-12.0.0	0.0172	114	212	403	238	213	309
	MSHB-13.0.0	0.0172	114	220	400	228	215	309
	MSHB-14.0.0	0.0172	117	212	400	232	215	309
	MSHB-15.0.0	0.0172	117	212	403	204	ND	307
	MSHB-16.0.0	0.0345	117	212	403	240	213	307
	MSHB-17.0.0	0.0172	117	216	400	234	213	307
	MSHB-18.0.0	0.0172	117	216	403	238	213	307
	MSHB-19.0.0	0.0172	117	216	403	238	213	309
	MSHB-20.0.0	0.0172	117	220	400	210	215	309
	MSHB-21.0.0	0.0214	117	220	400	228	215	307
	MSHB-22.0.0	0.0131	117	220	400	228	215	309
	MSHB-23.0.0	0.0172	117	220	400	228	ND	307
	MSHB-24.0.0	0.0172	117	220	400	234	217	309
	MSHB-25.0.0	0.0172	117	220	400	240	ND	309
	MSHB-26.0.0	0.0345	117	220	403	240	213	307
Yunnan wild boar	MSYWB-1.0.0	0.0263	117	200	403	218	215	307
	MSYWB-2.0.0	0.0263	117	204	400	224	211	307
	MSYWB-3.0.0	0.0263	117	204	400	234	213	307
	MSYWB-4.0.0	0.0263	117	204	403	216	209	309
	MSYWB-5.0.0	0.0263	117	212	397	228	211	309
	MSYWB-6.0.0	0.0263	117	212	397	236	211	307
	MSYWB-7.0.0	0.0263	117	212	400	214	213	309
	MSYWB-8.0.0	0.0263	117	212	400	236	215	307
	MSYWB-9.0.0	0.0263	117	212	403	234	213	315
	MSYWB-10.0.0	0.0263	117	212	403	236	211	309
	MSYWB-11.0.0	0.0263	117	212	403	236	213	309
	MSYWB-12.0.0	0.0263	117	216	397	228	217	307
	MSYWB-13.0.0	0.0263	117	216	397	234	217	315
	MSYWB-14.0.0	0.0263	117	216	400	220	211	309
	MSYWB-15.0.0	0.0263	117	216	400	220	213	315
	MSYWB-16.0.0	0.0263	117	216	400	224	211	307
	MSYWB-17.0.0	0.0263	117	216	400	248	211	315
	MSYWB-18.0.0	0.0263	117	216	403	226	209	309

	MSYWB-19.0.0	0.0263	117	216	403	228	211	307
	MSYWB-20.0.0	0.0263	117	216	403	230	211	315
	MSYWB-21.0.0	0.0263	117	216	403	238	217	307
	MSYWB-22.0.0	0.0263	117	216	403	240	211	307
	MSYWB-23.0.0	0.0263	117	220	400	224	209	309
	MSYWB-24.0.0	0.0263	117	220	400	224	213	315
	MSYWB-25.0.0	0.0263	117	220	400	230	211	307
	MSYWB-26.0.0	0.0263	117	220	403	216	215	309
	MSYWB-27.0.0	0.0263	117	220	403	242	213	309
	MSYWB-28.0.0	0.0263	117	232	403	236	217	309
	MSYWB-29.0.0	0.0526	ND	ND	403	ND	213	ND
Huanan	MSHWB-1.0.0	0.0156	111	220	400	224	215	327
wild boar	MSHWB-2.0.0	0.0156	111	220	403	226	217	309
	MSHWB-3.0.0	0.0469	111	220	403	232	213	307
	MSHWB-4.0.0	0.0156	111	220	406	ND	215	315
	MSHWB-5.0.0	0.0156	114	ND	ND	222	ND	309
	MSHWB-6.0.0	0.0156	117	212	391	248	213	309
	MSHWB-7.0.0	0.0156	117	212	397	ND	217	327
	MSHWB-8.0.0	0.0156	117	212	400	244	217	315
	MSHWB-9.0.0	0.0156	117	212	400	ND	213	303
	MSHWB-10.0.0	0.0156	117	212	403	230	215	307
	MSHWB-11.0.0	0.0156	117	212	403	232	215	329
	MSHWB-12.0.0	0.0156	117	216	400	232	219	307
	MSHWB-13.0.0	0.0156	117	216	403	244	213	307
	MSHWB-14.0.0	0.0156	117	220	400	230	215	307
	MSHWB-15.0.0	0.0156	117	220	400	230	215	309
	MSHWB-16.0.0	0.0156	117	220	400	232	213	309
	MSHWB-17.0.0	0.0156	117	220	400	232	213	327
	MSHWB-18.0.0	0.0156	117	220	400	234	213	327
	MSHWB-19.0.0	0.0156	117	220	400	234	215	309
	MSHWB-20.0.0	0.0156	117	220	400	234	215	319
	MSHWB-21.0.0	0.0156	117	220	400	234	217	309
	MSHWB-22.0.0	0.0156	117	220	400	236	219	309
	MSHWB-23.0.0	0.0156	117	220	400	244	213	311
	MSHWB-24.0.0	0.0156	117	220	400	244	213	317
	MSHWB-25.0.0	0.0156	117	220	400	252	211	319
	MSHWB-26.0.0	0.0156	117	220	403	224	211	319
	MSHWB-27.0.0	0.0469	117	220	403	224	213	307
	MSHWB-28.0.0	0.0469	117	220	403	224	213	309
	MSHWB-29.0.0	0.0156	117	220	403	224	213	327
	MSHWB-30.0.0	0.0156	117	220	403	224	217	307
	MSHWB-31.0.0	0.0156	117	220	403	226	213	309
	MSHWB-32.0.0	0.0156	117	220	403	230	215	309
	MSHWB-33.0.0	0.0156	117	220	403	232	213	309
	MSHWB-34.0.0	0.0156	117	220	403	232	215	329
	MSHWB-35.0.0	0.0156	117	220	403	232	217	307
	MSHWB-36.0.0	0.0156	117	220	403	232	227	327
	MSHWB-37.0.0	0.0156	117	220	403	234	215	311
	MSHWB-38.0.0	0.0313	117	220	403	236	213	307
	MSHWB-39.0.0	0.0156	117	220	403	236	215	309
	MSHWB-40.0.0	0.0156	117	220	403	236	215	311
	MSHWB-41.0.0	0.0156	117	220	403	236	219	307
	MSHWB-42.0.0	0.0156	117	220	403	246	213	307
	MSHWB-43.0.0	0.0156	117	220	403	246	213	327
	MSHWB-44.0.0	0.0313	117	220	403	ND	213	307
	MSHWB-45.0.0	0.0313	117	220	406	232	215	315
	MSHWB-46.0.0	0.0156	117	220	406	ND	213	303
	MSHWB-47.0.0	0.0156	117	ND	400	228	ND	309

	MSHWB-48.0.0	0.0156	117	ND	400	228	ND	317
	MSHWB-49.0.0	0.0156	117	ND	400	ND	211	309
	MSHWB-50.0.0	0.0156	117	ND	403	ND	219	307
	MSHWB-51.0.0	0.0156	117	ND	ND	248	ND	307
Dongbei wild boar	MSDWB-1.0.0	0.0313	111	212	400	228	215	309
	MSDWB-2.0.0	0.0451	111	212	400	230	215	319
	MSDWB-3.0.0	0.0156	111	212	400	232	217	307
	MSDWB-4.0.0	0.0156	111	212	400	234	217	307
	MSDWB-5.0.0	0.0156	111	216	400	234	217	309
	MSDWB-6.0.0	0.0174	111	220	403	230	215	319
	MSDWB-7.0.0	0.0313	111	220	403	252	211	319
	MSDWB-8.0.0	0.0156	111	224	400	228	215	319
	MSDWB-9.0.0	0.0313	117	212	400	228	215	309
	MSDWB-10.0.0	0.0174	117	212	400	230	213	307
	MSDWB-11.0.0	0.0156	117	212	403	210	215	315
	MSDWB-12.0.0	0.0156	117	212	403	230	213	303
	MSDWB-13.0.0	0.0156	117	212	403	230	215	319
	MSDWB-14.0.0	0.0156	117	212	403	232	215	319
	MSDWB-15.0.0	0.0156	117	216	400	230	215	327
	MSDWB-16.0.0	0.0156	117	216	400	230	217	309
	MSDWB-17.0.0	0.0156	117	216	400	230	227	309
	MSDWB-18.0.0	0.0156	117	216	400	234	215	309
	MSDWB-19.0.0	0.0156	117	216	403	226	213	309
	MSDWB-20.0.0	0.0156	117	220	400	226	213	309
	MSDWB-21.0.0	0.0469	117	220	400	232	215	303
	MSDWB-22.0.0	0.0469	117	220	400	232	217	303
	MSDWB-23.0.0	0.0156	117	220	400	234	215	319
	MSDWB-24.0.0	0.0156	117	220	400	234	215	327
	MSDWB-25.0.0	0.0156	117	220	400	234	217	307
	MSDWB-26.0.0	0.0156	117	220	403	230	213	319
	MSDWB-27.0.0	0.0313	117	220	406	244	213	303
	MSDWB-28.0.0	0.0156	117	224	400	228	217	309
	MSDWB-29.0.0	0.0156	117	224	403	230	215	307
	MSDWB-30.0.0	0.0156	117	224	403	252	211	319

#2 Rare haplotypes of SLA Class II region based on microsatellite typing (Frequency < 5%, ND: Not detected)

Population	Rare Haplotypes	Frequencies	Name of MS marker: SLAMS								
			047	092	048	049	050	051	052	095	094
Diannan small-ear pig	MSDN-0.1.0	0.0100	211	288	195	299	129	262	226	232	399
	MSDN-0.2.0	0.0100	211	290	195	289	131	262	220	232	391
	MSDN-0.3.0	0.0100	211	292	195	293	139	262	226	230	391
	MSDN-0.4.0	0.0100	211	292	213	287	125	262	230	228	391
	MSDN-0.5.0	0.0100	213	284	197	293	149	266	230	228	391
	MSDN-0.6.0	0.0100	213	292	183	295	133	262	236	232	391
	MSDN-0.7.0	0.0100	213	292	187	291	141	262	240	228	391
	MSDN-0.8.0	0.0100	213	292	195	293	119	262	218	228	391
	MSDN-0.9.0	0.0100	213	292	197	291	131	262	226	228	391
	MSDN-0.10.0	0.0100	213	292	197	293	133	262	226	232	391
	MSDN-0.11.0	0.0100	213	292	197	293	143	262	220	232	391
	MSDN-0.12.0	0.0100	213	292	197	293	155	262	252	230	387
	MSDN-0.13.0	0.0100	213	294	187	289	149	262	250	232	391
	MSDN-0.14.0	0.0100	213	298	185	289	121	262	228	228	391
	MSDN-0.15.0	0.0100	215	284	199	287	125	262	220	228	391
	MSDN-0.16.0	0.0100	215	288	187	289	119	262	230	226	ND
	MSDN-0.17.0	0.0100	215	288	187	289	153	262	ND	232	391
	MSDN-0.18.0	0.0100	215	288	201	293	125	258	220	232	391
	MSDN-0.19.0	0.0100	215	290	185	291	153	262	230	232	391
	MSDN-0.20.0	0.0100	215	290	195	297	135	262	220	232	391
	MSDN-0.21.0	0.0100	215	290	197	291	153	258	ND	228	391
	MSDN-0.22.0	0.0100	215	292	185	289	119	262	230	228	391
	MSDN-0.23.0	0.0100	215	292	185	289	121	258	236	228	391
	MSDN-0.24.0	0.0100	215	292	187	293	135	262	224	228	391
	MSDN-0.25.0	0.0100	215	292	197	293	127	262	226	232	391
	MSDN-0.26.0	0.0100	215	294	199	291	131	266	248	228	391
	MSDN-0.27.0	0.0100	215	296	187	289	137	262	220	232	391
	MSDN-0.28.0	0.0100	219	282	197	293	137	258	226	220	391
	MSDN-0.29.0	0.0100	219	294	187	289	129	258	224	232	391
	MSDN-0.30.0	0.0100	219	298	213	287	119	258	220	228	391
	MSDN-0.31.0	0.0100	223	282	197	293	119	258	236	230	391
	MSDN-0.32.0	0.0100	227	292	187	293	141	258	220	232	391
	MSDN-0.33.0	0.0100	229	282	187	291	153	266	232	232	391
	MSDN-0.34.0	0.0100	229	296	203	295	133	262	242	230	391
	MSDN-0.35.0	0.0100	231	282	187	287	127	262	238	232	391
	MSDN-0.36.0	0.0100	231	282	187	291	153	262	232	230	391
	MSDN-0.37.0	0.0100	231	282	195	295	125	266	238	232	391
	MSDN-0.38.0	0.0100	231	284	203	291	133	266	232	230	391
	MSDN-0.39.0	0.0100	231	288	195	293	121	262	228	230	391
	MSDN-0.40.0	0.0100	231	290	187	291	119	262	230	230	391
	MSDN-0.41.0	0.0100	231	292	183	295	131	258	224	232	383
	MSDN-0.42.0	0.0100	231	292	187	293	153	262	220	232	391
	MSDN-0.43.0	0.0100	231	292	187	293	155	266	238	230	391
	MSDN-0.44.0	0.0100	231	292	201	293	141	262	230	230	391
	MSDN-0.45.0	0.0100	231	294	185	297	141	262	220	230	391
	MSDN-0.46.0	0.0100	231	294	193	289	123	258	236	238	391
	MSDN-0.47.0	0.0100	233	282	193	287	131	266	232	228	391
	MSDN-0.48.0	0.0100	233	284	187	289	131	262	234	228	391
	MSDN-0.49.0	0.0100	233	288	185	293	133	262	228	228	391
	MSDN-0.50.0	0.0100	233	288	187	285	121	262	230	226	ND
	MSDN-0.51.0	0.0100	233	288	193	297	119	262	224	214	391
	MSDN-0.52.0	0.0100	233	288	205	293	127	258	224	228	391
	MSDN-0.53.0	0.0100	233	290	183	295	129	262	246	230	391

	MSDN-0.54.0	0.0100	233	290	197	289	119	262	232	230	391
	MSDN-0.55.0	0.0100	233	292	187	287	133	262	246	228	391
	MSDN-0.56.0	0.0100	233	292	195	295	121	262	244	230	391
	MSDN-0.57.0	0.0100	233	294	187	289	121	262	224	232	391
	MSDN-0.58.0	0.0100	233	294	193	287	119	266	258	232	391
	MSDN-0.59.0	0.0100	233	294	193	289	131	266	230	232	391
	MSDN-0.60.0	0.0100	235	282	185	289	131	258	232	228	391
	MSDN-0.61.0	0.0100	235	284	195	291	131	266	230	232	391
	MSDN-0.62.0	0.0100	235	284	211	293	157	262	248	236	387
	MSDN-0.63.0	0.0100	235	288	195	289	133	258	232	232	387
	MSDN-0.64.0	0.0100	235	292	193	289	157	266	234	232	391
	MSDN-0.65.0	0.0100	235	292	203	287	119	266	230	230	387
	MSDN-0.66.0	0.0100	235	294	193	289	131	262	232	232	387
	MSDN-0.67.0	0.0100	237	282	187	293	125	262	224	232	391
	MSDN-0.68.0	0.0100	237	282	195	289	159	258	250	232	391
	MSDN-0.69.0	0.0100	237	284	193	287	125	266	232	232	387
	MSDN-0.70.0	0.0100	237	284	193	289	121	266	248	228	391
	MSDN-0.71.0	0.0100	237	288	183	293	119	262	232	232	383
	MSDN-0.72.0	0.0100	237	288	203	295	125	262	242	232	391
	MSDN-0.73.0	0.0100	237	290	185	289	125	270	232	238	391
	MSDN-0.74.0	0.0100	237	290	187	295	157	266	228	230	387
	MSDN-0.75.0	0.0100	237	290	195	293	155	262	234	230	391
	MSDN-0.76.0	0.0100	237	290	197	291	157	266	228	230	387
	MSDN-0.77.0	0.0100	237	290	201	293	155	258	220	232	391
	MSDN-0.78.0	0.0100	237	292	185	285	141	266	230	230	391
	MSDN-0.79.0	0.0100	237	292	185	289	153	258	230	228	391
	MSDN-0.80.0	0.0100	237	292	185	295	131	270	214	230	391
	MSDN-0.81.0	0.0100	237	292	187	293	137	266	230	230	387
	MSDN-0.82.0	0.0200	237	292	187	297	133	266	232	230	391
	MSDN-0.83.0	0.0100	237	292	193	289	119	262	234	230	391
	MSDN-0.84.0	0.0100	237	292	193	289	119	266	230	230	391
	MSDN-0.85.0	0.0100	237	292	203	293	147	262	236	230	391
	MSDN-0.86.0	0.0100	237	292	205	293	153	262	230	230	391
	MSDN-0.87.0	0.0100	237	294	187	293	133	266	232	230	391
	MSDN-0.88.0	0.0100	239	282	195	293	125	266	234	228	391
	MSDN-0.89.0	0.0100	239	284	185	289	131	262	246	232	391
	MSDN-0.90.0	0.0100	239	284	193	289	131	258	232	232	391
	MSDN-0.91.0	0.0100	239	286	185	295	131	262	220	230	391
	MSDN-0.92.0	0.0100	239	286	199	289	119	258	234	228	391
	MSDN-0.93.0	0.0100	239	288	185	289	129	262	230	230	399
	MSDN-0.94.0	0.0100	239	290	187	293	131	262	232	232	391
	MSDN-0.95.0	0.0100	239	292	187	295	137	262	232	230	391
	MSDN-0.96.0	0.0100	239	294	187	291	151	258	230	232	391
	MSDN-0.97.0	0.0100	239	294	187	293	125	258	242	232	391
	MSDN-0.98.0	0.0100	239	298	199	291	153	262	218	228	391
	MSDN-0.99.0	0.0100	241	288	205	289	131	262	246	228	387
Congjiang Xiang pig	MSCJX-0.1.0	0.0143	215	288	195	289	121	266	220	228	391
	MSCJX-0.2.0	0.0143	215	288	211	293	131	266	228	244	391
	MSCJX-0.3.0	0.0143	215	290	191	293	153	258	226	230	391
	MSCJX-0.4.0	0.0286	215	294	195	289	145	266	220	228	391
	MSCJX-0.5.0	0.0143	215	294	195	289	145	266	220	230	391
	MSCJX-0.6.0	0.0143	215	298	211	289	135	262	212	228	391
	MSCJX-0.7.0	0.0143	221	286	195	297	155	266	236	230	391
	MSCJX-0.8.0	0.0143	221	294	195	297	121	262	232	230	391
	MSCJX-0.9.0	0.0143	221	294	203	295	155	262	226	230	391
	MSCJX-0.10.0	0.0143	221	296	199	289	131	266	232	232	391
	MSCJX-0.11.0	0.0143	221	ND	209	291	131	266	232	242	391
	MSCJX-0.12.0	0.0143	231	282	195	293	129	266	234	228	391

	MSCJX-0.13.0	0.0286	231	284	195	295	145	266	220	228	391
	MSCJX-0.14.0	0.0143	231	294	199	297	125	266	228	230	391
	MSCJX-0.15.0	0.0143	231	294	199	297	127	266	220	234	391
	MSCJX-0.16.0	0.0143	231	296	197	295	145	262	234	220	391
	MSCJX-0.17.0	0.0143	233	282	195	295	145	266	232	212	391
	MSCJX-0.18.0	0.0143	233	284	195	287	145	266	234	232	391
	MSCJX-0.19.0	0.0143	233	284	195	289	147	266	220	234	391
	MSCJX-0.20.0	0.0143	233	284	195	295	145	266	232	234	391
	MSCJX-0.21.0	0.0143	233	298	197	289	145	266	220	212	391
	MSCJX-0.22.0	0.0143	235	288	195	295	159	266	232	228	391
	MSCJX-0.23.0	0.0143	235	292	203	297	155	266	234	228	391
	MSCJX-0.24.0	0.0143	235	292	207	287	131	266	220	228	391
	MSCJX-0.25.0	0.0143	235	296	185	289	147	266	220	228	391
	MSCJX-0.26.0	0.0143	235	296	195	291	155	258	226	228	391
	MSCJX-0.27.0	0.0143	235	296	197	295	137	266	232	230	391
	MSCJX-0.28.0	0.0143	235	296	203	295	155	266	232	228	391
	MSCJX-0.29.0	0.0143	235	296	203	295	ND	266	234	228	391
	MSCJX-0.30.0	0.0286	235	298	195	297	143	258	232	230	391
	MSCJX-0.31.0	0.0286	235	298	197	295	143	266	232	214	391
	MSCJX-0.32.0	0.0143	235	298	211	293	141	266	220	228	391
	MSCJX-0.33.0	0.0143	237	282	195	287	145	262	220	228	391
	MSCJX-0.34.0	0.0143	237	282	195	289	127	266	228	234	391
	MSCJX-0.35.0	0.0143	237	282	195	289	145	266	220	242	391
	MSCJX-0.36.0	0.0143	237	282	197	287	129	258	220	228	391
	MSCJX-0.37.0	0.0286	237	284	195	289	145	266	232	244	391
	MSCJX-0.38.0	0.0143	237	284	195	299	129	266	220	232	391
	MSCJX-0.39.0	0.0143	237	284	197	289	141	258	220	228	391
	MSCJX-0.40.0	0.0143	237	284	197	ND	145	258	232	228	391
	MSCJX-0.41.0	0.0143	237	288	195	289	159	262	232	228	391
	MSCJX-0.42.0	0.0143	237	288	211	293	131	266	236	228	391
	MSCJX-0.43.0	0.0143	237	292	195	ND	155	266	220	244	391
	MSCJX-0.44.0	0.0286	237	292	197	293	155	258	220	228	391
	MSCJX-0.45.0	0.0143	237	292	197	297	155	266	218	234	391
	MSCJX-0.46.0	0.0143	237	296	195	297	155	262	220	228	391
	MSCJX-0.47.0	0.0143	237	296	203	287	155	266	234	230	391
	MSCJX-0.48.0	0.0143	237	298	211	293	131	266	220	230	391
	MSCJX-0.49.0	0.0143	237	ND	209	291	131	266	220	242	391
	MSCJX-0.50.0	0.0143	239	282	203	289	145	262	234	228	391
	MSCJX-0.51.0	0.0143	239	292	185	295	145	266	220	230	391
	MSCJX-0.52.0	0.0143	239	292	195	293	155	266	220	230	391
	MSCJX-0.53.0	0.0143	239	296	203	295	155	266	234	228	391
	MSCJX-0.54.0	0.0143	239	296	203	297	129	258	234	228	391
	MSCJX-0.55.0	0.0286	239	296	203	297	155	266	234	228	391
	MSCJX-0.56.0	0.0143	239	298	197	289	143	266	232	228	391
	MSCJX-0.57.0	0.0143	239	298	197	295	ND	266	232	228	391
	MSCJX-0.58.0	0.0143	239	298	203	295	137	266	234	228	391
	MSCJX-0.59.0	0.0143	239	306	203	297	155	266	234	228	391
Bama miniature pig	MSBM-0.1.0	0.0156	215	282	197	295	141	258	234	232	391
	MSBM-0.2.0	0.0156	215	284	197	289	131	266	212	228	391
	MSBM-0.3.0	0.0156	215	284	197	297	131	262	224	232	391
	MSBM-0.4.0	0.0156	215	290	195	291	141	258	224	230	391
	MSBM-0.5.0	0.0313	215	304	197	297	141	258	224	230	391
	MSBM-0.6.0	0.0156	225	292	197	293	129	266	226	228	391
	MSBM-0.7.0	0.0156	227	288	195	287	135	266	234	228	391
	MSBM-0.8.0	0.0156	227	288	195	287	159	258	234	230	391
	MSBM-0.9.0	0.0156	229	282	195	289	141	266	232	228	391
	MSBM-0.10.0	0.0156	231	290	193	289	129	258	238	230	391
	MSBM-0.11.0	0.0156	233	288	199	289	151	270	234	232	391

	MSBM-0.12.0	0.0156	233	288	213	287	129	258	228	230	391
	MSBM-0.13.0	0.0156	233	290	195	291	141	258	238	228	391
	MSBM-0.14.0	0.0156	233	290	195	297	141	266	232	228	391
	MSBM-0.15.0	0.0156	233	292	195	291	135	262	238	230	391
	MSBM-0.16.0	0.0156	233	292	195	291	141	262	234	232	391
	MSBM-0.17.0	0.0156	233	292	213	297	141	266	234	230	391
	MSBM-0.18.0	0.0156	233	296	193	289	129	262	238	228	391
	MSBM-0.19.0	0.0156	233	298	197	297	141	270	234	228	391
	MSBM-0.20.0	0.0156	235	282	199	315	125	262	238	232	391
	MSBM-0.21.0	0.0156	235	290	193	295	141	262	236	230	391
	MSBM-0.22.0	0.0156	235	290	195	295	141	262	236	230	391
	MSBM-0.23.0	0.0156	235	294	195	289	135	266	234	228	391
	MSBM-0.24.0	0.0156	235	294	195	289	141	266	234	232	391
	MSBM-0.25.0	0.0156	235	294	195	289	141	266	242	232	391
	MSBM-0.26.0	0.0313	235	296	199	315	125	262	238	232	391
	MSBM-0.27.0	0.0156	235	298	195	289	125	270	226	232	391
	MSBM-0.28.0	0.0156	235	298	195	295	153	258	224	228	391
	MSBM-0.29.0	0.0156	237	282	193	289	141	266	232	228	391
	MSBM-0.30.0	0.0156	237	282	193	289	141	266	238	230	391
	MSBM-0.31.0	0.0156	237	282	195	287	129	266	232	232	391
	MSBM-0.32.0	0.0156	237	282	195	289	141	258	232	232	391
	MSBM-0.33.0	0.0156	237	282	195	289	141	258	238	228	391
	MSBM-0.34.0	0.0313	237	282	195	289	141	266	238	228	391
	MSBM-0.35.0	0.0156	237	282	199	297	141	270	232	232	391
	MSBM-0.36.0	0.0156	237	284	195	291	129	266	234	228	391
	MSBM-0.37.0	0.0156	237	290	195	289	129	266	232	232	391
	MSBM-0.38.0	0.0156	237	290	195	291	141	266	238	228	391
	MSBM-0.39.0	0.0156	237	292	195	287	133	266	220	232	391
	MSBM-0.40.0	0.0156	237	296	199	295	145	266	212	240	391
	MSBM-0.41.0	0.0313	237	298	195	289	129	266	232	228	391
	MSBM-0.42.0	0.0156	237	298	195	295	141	270	232	242	391
	MSBM-0.43.0	0.0156	237	298	197	295	141	270	240	228	391
	MSBM-0.44.0	0.0156	237	298	199	295	141	266	240	228	391
	MSBM-0.45.0	0.0156	237	298	199	297	141	266	232	228	391
	MSBM-0.46.0	0.0156	237	300	211	291	141	258	234	230	391
	MSBM-0.47.0	0.0156	239	282	195	289	141	258	234	244	391
	MSBM-0.48.0	0.0156	239	282	195	295	141	266	238	228	391
	MSBM-0.49.0	0.0156	239	282	197	289	141	258	238	232	391
	MSBM-0.50.0	0.0156	239	282	197	295	147	266	218	228	391
	MSBM-0.51.0	0.0156	239	292	185	287	141	266	220	228	391
	MSBM-0.52.0	0.0156	239	292	195	293	141	266	228	228	391
	MSBM-0.53.0	0.0156	239	292	199	295	125	262	240	228	391
	MSBM-0.54.0	0.0156	239	292	199	299	149	266	220	228	391
	MSBM-0.55.0	0.0156	239	300	197	295	135	262	238	230	391
	MSBM-0.56.0	0.0156	241	282	197	293	141	266	228	228	391
	MSBM-0.57.0	0.0156	241	288	185	287	155	266	220	228	391
	MSBM-0.58.0	0.0156	241	288	201	297	145	262	234	228	391
	MSBM-0.59.0	0.0156	241	294	197	295	141	266	224	230	391
	MSBM-0.60.0	0.0156	241	298	199	297	141	270	234	228	391
Wuzhishan pig	MSWZS-0.1.0	0.0156	211	284	185	289	133	ND	243	228	391
	MSWZS-0.2.0	0.0156	211	290	185	291	125	274	228	232	391
	MSWZS-0.3.0	0.0313	211	292	193	291	125	262	228	232	391
	MSWZS-0.4.0	0.0156	211	292	201	295	159	266	236	230	391
	MSWZS-0.5.0	0.0156	211	294	185	289	133	266	240	230	391
	MSWZS-0.6.0	0.0156	211	294	195	297	125	266	238	244	391
	MSWZS-0.7.0	0.0156	211	294	197	295	157	258	238	244	391
	MSWZS-0.8.0	0.0156	211	298	197	291	153	266	228	228	391
	MSWZS-0.9.0	0.0156	211	298	197	297	155	266	242	228	391

	MSWZS-0.10.0	0.0156	211	298	197	297	157	274	242	232	391
	MSWZS-0.11.0	0.0156	215	282	193	295	145	262	232	230	391
	MSWZS-0.12.0	0.0156	215	286	197	289	133	266	218	232	391
	MSWZS-0.13.0	0.0156	215	286	197	289	133	266	238	228	391
	MSWZS-0.14.0	0.0156	215	288	197	291	125	270	236	228	391
	MSWZS-0.15.0	0.0156	215	288	197	295	133	266	236	242	391
	MSWZS-0.16.0	0.0156	215	290	193	285	141	262	226	246	391
	MSWZS-0.17.0	0.0156	215	290	193	291	125	262	228	230	391
	MSWZS-0.18.0	0.0156	215	290	195	291	125	262	228	242	391
	MSWZS-0.19.0	0.0156	215	290	195	295	125	274	220	230	391
	MSWZS-0.20.0	0.0156	215	290	195	295	155	266	242	232	391
	MSWZS-0.21.0	0.0156	215	290	197	291	133	274	242	242	391
	MSWZS-0.22.0	0.0156	215	290	197	315	ND	ND	212	234	ND
	MSWZS-0.23.0	0.0156	215	290	207	297	157	260	226	228	391
	MSWZS-0.24.0	0.0156	215	292	195	289	125	274	236	230	391
	MSWZS-0.25.0	0.0156	215	294	193	291	125	262	226	242	391
	MSWZS-0.26.0	0.0156	215	294	195	295	127	274	240	244	391
	MSWZS-0.27.0	0.0156	215	294	195	295	155	ND	238	234	391
	MSWZS-0.28.0	0.0313	215	294	195	295	159	262	236	232	391
	MSWZS-0.29.0	0.0156	215	294	195	295	159	262	236	242	391
	MSWZS-0.30.0	0.0156	215	294	197	289	133	ND	240	226	391
	MSWZS-0.31.0	0.0156	215	294	197	315	127	262	242	234	391
	MSWZS-0.32.0	0.0156	215	296	195	295	123	ND	220	228	391
	MSWZS-0.33.0	0.0156	215	296	201	295	123	ND	226	230	391
	MSWZS-0.34.0	0.0156	215	298	193	315	155	266	228	234	395
	MSWZS-0.35.0	0.0156	215	298	195	295	157	266	244	230	391
	MSWZS-0.36.0	0.0156	231	296	195	291	125	266	238	228	391
	MSWZS-0.37.0	0.0156	233	286	197	289	157	266	238	230	391
	MSWZS-0.38.0	0.0156	233	286	197	295	157	270	244	228	391
	MSWZS-0.39.0	0.0156	233	288	197	289	133	270	244	228	391
	MSWZS-0.40.0	0.0156	233	288	197	291	157	274	236	232	391
	MSWZS-0.41.0	0.0156	233	288	197	297	141	266	220	242	391
	MSWZS-0.42.0	0.0156	233	290	185	289	155	266	238	234	391
	MSWZS-0.43.0	0.0156	233	290	195	291	157	262	226	242	391
	MSWZS-0.44.0	0.0156	233	292	193	285	141	262	238	228	391
	MSWZS-0.45.0	0.0156	233	292	195	291	125	262	236	246	391
	MSWZS-0.46.0	0.0156	233	294	185	295	133	ND	220	228	391
	MSWZS-0.47.0	0.0156	235	290	195	295	127	262	212	230	391
	MSWZS-0.48.0	0.0156	235	290	197	315	159	262	212	228	391
	MSWZS-0.49.0	0.0156	235	294	195	295	ND	ND	218	246	ND
	MSWZS-0.50.0	0.0156	237	284	195	295	159	262	226	228	391
	MSWZS-0.51.0	0.0156	237	294	185	289	133	266	240	228	391
	MSWZS-0.52.0	0.0156	237	294	195	289	133	266	220	232	391
	MSWZS-0.53.0	0.0156	239	294	185	295	133	266	220	228	391
	MSWZS-0.54.0	0.0156	241	282	195	287	151	ND	220	232	391
	MSWZS-0.55.0	0.0156	241	290	197	291	125	270	244	228	391
	MSWZS-0.56.0	0.0156	241	294	185	291	157	266	220	230	391
	MSWZS-0.57.0	0.0156	241	294	195	295	155	266	220	228	391
	MSWZS-0.58.0	0.0156	241	294	195	295	155	ND	258	224	391
	MSWZS-0.59.0	0.0156	241	294	195	295	159	266	220	230	391
	MSWZS-0.60.0	0.0156	241	294	199	287	151	ND	226	226	391
	MSWZS-0.61.0	0.0156	241	296	191	295	133	ND	238	228	391
	MSWZS-0.62.0	0.0156	241	296	195	295	155	266	236	242	391
Mingguang small-ear pig	MSMG-0.1.0	0.0100	207	284	193	293	133	266	232	232	391
	MSMG-0.2.0	0.0100	207	284	209	293	123	266	228	228	391
	MSMG-0.3.0	0.0100	207	286	195	291	127	ND	236	232	391
	MSMG-0.4.0	0.0100	211	280	195	293	123	262	228	230	391
	MSMG-0.5.0	0.0100	211	282	185	293	137	262	238	230	391

MSMG-0.6.0	0.0100	211	286	195	295	133	262	230	230	391
MSMG-0.7.0	0.0100	211	286	199	293	121	262	226	228	391
MSMG-0.8.0	0.0100	211	292	195	289	125	262	226	230	391
MSMG-0.9.0	0.0100	211	294	201	283	133	258	218	232	391
MSMG-0.10.0	0.0100	213	282	185	293	139	ND	236	228	391
MSMG-0.11.0	0.0100	213	290	215	291	123	258	240	228	391
MSMG-0.12.0	0.0100	213	292	191	295	135	258	228	232	391
MSMG-0.13.0	0.0100	213	294	195	293	159	ND	232	228	391
MSMG-0.14.0	0.0100	213	ND	195	293	ND	ND	228	230	395
MSMG-0.15.0	0.0100	215	280	203	295	131	262	228	232	391
MSMG-0.16.0	0.0100	215	280	207	287	129	ND	232	230	391
MSMG-0.17.0	0.0100	215	282	203	295	133	262	242	230	391
MSMG-0.18.0	0.0100	215	286	193	293	123	258	232	228	391
MSMG-0.19.0	0.0100	215	288	187	289	121	ND	232	228	391
MSMG-0.20.0	0.0100	215	288	193	285	121	262	258	228	391
MSMG-0.21.0	0.0100	215	288	193	287	131	262	232	228	391
MSMG-0.22.0	0.0100	215	288	207	293	133	262	238	230	391
MSMG-0.23.0	0.0100	215	288	211	ND	129	258	228	226	391
MSMG-0.24.0	0.0100	215	290	195	293	157	262	226	228	391
MSMG-0.25.0	0.0100	215	290	199	293	121	ND	248	228	395
MSMG-0.26.0	0.0100	215	290	205	287	125	266	230	228	391
MSMG-0.27.0	0.0100	215	292	195	293	123	262	220	228	391
MSMG-0.28.0	0.0100	215	292	195	293	133	262	246	230	391
MSMG-0.29.0	0.0100	215	292	195	295	151	262	230	230	391
MSMG-0.30.0	0.0100	215	292	201	295	119	262	242	232	391
MSMG-0.31.0	0.0100	215	294	199	293	121	ND	232	228	391
MSMG-0.32.0	0.0100	215	296	201	293	119	258	230	232	391
MSMG-0.33.0	0.0100	215	296	201	293	123	258	230	232	391
MSMG-0.34.0	0.0100	215	298	195	285	129	262	244	230	391
MSMG-0.35.0	0.0100	215	300	195	287	131	ND	232	230	391
MSMG-0.36.0	0.0100	215	300	203	287	119	ND	220	230	391
MSMG-0.37.0	0.0100	221	286	185	293	123	258	236	230	391
MSMG-0.38.0	0.0100	221	296	199	289	125	ND	212	232	391
MSMG-0.39.0	0.0100	223	284	197	293	119	258	230	230	391
MSMG-0.40.0	0.0100	223	290	195	283	125	262	234	228	391
MSMG-0.41.0	0.0100	227	280	211	293	133	262	236	230	391
MSMG-0.42.0	0.0100	227	282	203	293	133	262	246	230	391
MSMG-0.43.0	0.0100	227	286	185	289	133	258	244	230	391
MSMG-0.44.0	0.0100	227	288	199	291	131	ND	218	228	391
MSMG-0.45.0	0.0100	227	290	195	293	121	262	228	228	391
MSMG-0.46.0	0.0100	227	290	205	289	119	ND	230	228	391
MSMG-0.47.0	0.0100	227	292	185	295	137	262	218	232	391
MSMG-0.48.0	0.0100	227	292	195	303	123	262	230	232	391
MSMG-0.49.0	0.0100	227	292	195	303	127	262	230	228	391
MSMG-0.50.0	0.0100	229	284	197	287	141	262	224	228	391
MSMG-0.51.0	0.0100	231	284	189	289	133	266	232	232	391
MSMG-0.52.0	0.0100	231	284	195	293	119	262	232	228	391
MSMG-0.53.0	0.0100	231	284	203	291	123	262	248	232	391
MSMG-0.54.0	0.0100	231	286	197	289	131	258	230	228	391
MSMG-0.55.0	0.0100	231	286	199	287	127	258	232	232	391
MSMG-0.56.0	0.0100	231	288	187	291	127	262	248	228	391
MSMG-0.57.0	0.0100	231	288	195	293	131	262	228	230	391
MSMG-0.58.0	0.0100	231	290	193	289	129	262	228	228	391
MSMG-0.59.0	0.0100	231	290	195	287	131	ND	232	228	395
MSMG-0.60.0	0.0100	231	290	195	293	119	258	220	228	391
MSMG-0.61.0	0.0100	231	290	203	291	123	262	228	232	395
MSMG-0.62.0	0.0100	231	292	201	295	119	258	226	228	391
MSMG-0.63.0	0.0100	233	286	195	295	123	262	232	232	391

	MSMG-0.64.0	0.0100	233	288	203	295	121	262	234	232	391
	MSMG-0.65.0	0.0100	233	290	203	285	133	262	236	230	391
	MSMG-0.66.0	0.0100	233	290	209	289	123	ND	232	228	391
	MSMG-0.67.0	0.0100	233	292	193	289	151	262	226	228	391
	MSMG-0.68.0	0.0100	233	292	197	289	155	258	220	232	391
	MSMG-0.69.0	0.0100	233	294	197	293	121	262	242	230	391
	MSMG-0.70.0	0.0100	235	282	193	289	131	266	228	230	391
	MSMG-0.71.0	0.0100	235	282	193	289	131	266	246	232	391
	MSMG-0.72.0	0.0100	235	282	193	295	129	266	232	232	391
	MSMG-0.73.0	0.0100	235	282	203	285	133	ND	212	232	391
	MSMG-0.74.0	0.0100	235	288	197	293	129	258	236	230	391
	MSMG-0.75.0	0.0100	235	292	195	289	119	258	234	228	391
	MSMG-0.76.0	0.0100	235	292	195	293	129	262	258	228	391
	MSMG-0.77.0	0.0100	235	296	187	289	131	258	220	228	391
	MSMG-0.78.0	0.0100	237	284	197	287	123	258	256	232	391
	MSMG-0.79.0	0.0100	237	286	187	ND	123	262	218	226	391
	MSMG-0.80.0	0.0100	237	288	201	293	123	262	234	232	391
	MSMG-0.81.0	0.0100	237	290	183	293	119	262	226	232	391
	MSMG-0.82.0	0.0100	237	292	197	293	133	266	230	230	391
	MSMG-0.83.0	0.0100	237	296	201	295	123	262	228	232	391
	MSMG-0.84.0	0.0100	237	ND	195	285	ND	ND	228	230	395
	MSMG-0.85.0	0.0200	239	282	185	293	119	258	242	230	391
	MSMG-0.86.0	0.0100	239	282	199	295	131	266	232	228	391
	MSMG-0.87.0	0.0100	239	284	195	293	131	ND	248	228	391
	MSMG-0.88.0	0.0100	239	284	197	291	123	262	236	230	391
	MSMG-0.89.0	0.0100	239	284	197	293	151	262	232	230	391
	MSMG-0.90.0	0.0100	239	286	195	295	127	262	228	230	391
	MSMG-0.91.0	0.0100	239	286	199	291	125	266	232	228	391
	MSMG-0.92.0	0.0100	239	286	203	291	121	ND	220	230	391
	MSMG-0.93.0	0.0100	239	286	209	295	119	ND	238	232	391
	MSMG-0.94.0	0.0100	239	288	193	285	141	262	220	228	391
	MSMG-0.95.0	0.0100	239	290	195	287	123	258	218	228	391
	MSMG-0.96.0	0.0100	239	290	199	295	153	270	228	228	395
	MSMG-0.97.0	0.0100	239	292	195	293	123	262	218	240	391
	MSMG-0.98.0	0.0100	239	292	201	295	139	258	230	228	391
	MSMG-0.99.0	0.0100	239	296	187	291	121	258	232	232	391
Hezuo pig	MSHZ-0.1.0	0.0143	215	284	197	303	119	262	224	230	391
	MSHZ-0.2.0	0.0143	215	284	205	295	119	258	234	228	391
	MSHZ-0.3.0	0.0143	215	284	205	295	129	262	232	232	391
	MSHZ-0.4.0	0.0143	215	284	205	295	155	262	236	228	391
	MSHZ-0.5.0	0.0143	215	288	197	295	133	262	226	ND	ND
	MSHZ-0.6.0	0.0143	215	290	199	295	125	266	234	228	391
	MSHZ-0.7.0	0.0286	215	298	197	303	119	266	234	230	391
	MSHZ-0.8.0	0.0143	215	298	201	287	125	266	234	244	391
	MSHZ-0.9.0	0.0143	215	298	201	293	125	266	218	244	ND
	MSHZ-0.10.0	0.0143	223	292	197	297	125	262	226	228	387
	MSHZ-0.11.0	0.0143	225	280	197	297	153	262	220	228	391
	MSHZ-0.12.0	0.0143	225	292	197	293	119	258	218	228	387
	MSHZ-0.13.0	0.0143	225	292	197	297	141	258	218	228	387
	MSHZ-0.14.0	0.0143	225	292	197	297	141	262	226	228	387
	MSHZ-0.15.0	0.0143	225	298	201	289	125	262	216	242	387
	MSHZ-0.16.0	0.0143	231	282	189	297	119	262	224	228	391
	MSHZ-0.17.0	0.0143	233	284	197	295	119	262	234	228	391
	MSHZ-0.18.0	0.0143	233	284	197	295	157	262	236	228	391
	MSHZ-0.19.0	0.0143	235	284	205	297	155	262	234	232	391
	MSHZ-0.20.0	0.0143	235	292	197	289	129	262	224	228	391
	MSHZ-0.21.0	0.0143	235	292	197	291	149	266	232	232	ND
	MSHZ-0.22.0	0.0143	235	296	187	291	119	ND	234	228	391

	MSHZ-0.23.0	0.0143	235	296	209	293	119	ND	234	230	391
	MSHZ-0.24.0	0.0143	235	298	189	289	125	262	234	240	ND
	MSHZ-0.25.0	0.0143	237	284	189	295	119	266	224	230	391
	MSHZ-0.26.0	0.0143	237	284	193	289	131	270	234	232	391
	MSHZ-0.27.0	0.0143	237	284	195	289	131	270	218	242	391
	MSHZ-0.28.0	0.0143	237	288	197	287	151	258	232	228	391
	MSHZ-0.29.0	0.0143	237	298	207	287	125	ND	212	232	391
	MSHZ-0.30.0	0.0143	239	284	213	295	125	262	220	228	387
	MSHZ-0.31.0	0.0143	239	288	201	287	125	ND	212	232	391
	MSHZ-0.32.0	0.0143	239	298	193	289	125	266	220	242	391
	MSHZ-0.33.0	0.0286	239	298	199	289	125	266	220	232	391
	MSHZ-0.34.0	0.0143	239	298	201	289	119	266	220	232	391
	MSHZ-0.35.0	0.0143	239	298	201	289	125	266	234	232	391
	MSHZ-0.36.0	0.0429	241	280	199	293	119	262	234	228	391
	MSHZ-0.37.0	0.0143	241	280	199	293	141	258	220	228	391
	MSHZ-0.38.0	0.0143	241	284	195	293	139	262	218	230	391
	MSHZ-0.39.0	0.0143	241	284	197	293	119	266	232	228	391
	MSHZ-0.40.0	0.0143	241	284	197	293	139	266	232	228	391
	MSHZ-0.41.0	0.0143	241	284	197	295	119	ND	234	232	391
	MSHZ-0.42.0	0.0143	241	284	199	289	151	262	232	ND	ND
	MSHZ-0.43.0	0.0143	241	284	203	287	129	270	234	230	391
	MSHZ-0.44.0	0.0143	241	288	187	295	119	262	224	232	391
	MSHZ-0.45.0	0.0143	241	288	197	295	119	ND	234	242	391
	MSHZ-0.46.0	0.0143	241	288	197	295	129	270	234	232	391
	MSHZ-0.47.0	0.0143	241	288	197	297	119	262	236	228	391
	MSHZ-0.48.0	0.0143	241	288	197	297	151	262	220	242	391
	MSHZ-0.49.0	0.0143	241	288	207	297	135	258	238	232	391
	MSHZ-0.50.0	0.0143	241	290	187	289	125	266	218	230	391
	MSHZ-0.51.0	0.0143	241	290	195	295	141	262	234	242	391
	MSHZ-0.52.0	0.0143	241	292	195	293	153	258	226	228	387
	MSHZ-0.53.0	0.0143	241	292	195	295	153	266	232	230	391
	MSHZ-0.54.0	0.0143	241	292	197	293	119	266	234	232	391
	MSHZ-0.55.0	0.0143	241	292	197	293	151	258	234	228	391
	MSHZ-0.56.0	0.0286	241	292	197	293	153	258	218	228	391
	MSHZ-0.57.0	0.0143	241	296	187	293	153	258	218	228	ND
	MSHZ-0.58.0	0.0286	241	296	193	289	119	262	234	242	391
	MSHZ-0.59.0	0.0143	241	296	197	289	141	262	234	228	391
	MSHZ-0.60.0	0.0143	241	300	195	293	129	258	218	240	391
Hebao pig	MSHB-0.1.0	0.0172	215	284	197	287	131	266	220	228	391
	MSHB-0.2.0	0.0172	215	290	199	297	119	266	238	228	391
	MSHB-0.3.0	0.0172	215	294	201	297	121	266	224	232	391
	MSHB-0.4.0	0.0172	215	296	185	287	119	266	240	228	391
	MSHB-0.5.0	0.0172	215	296	185	297	153	262	218	228	391
	MSHB-0.6.0	0.0172	215	296	201	297	119	266	212	228	391
	MSHB-0.7.0	0.0172	215	296	201	297	119	266	234	228	391
	MSHB-0.8.0	0.0172	215	296	201	297	153	262	220	228	391
	MSHB-0.9.0	0.0172	215	296	201	297	ND	266	234	228	387
	MSHB-0.10.0	0.0345	215	298	185	287	119	266	220	230	391
	MSHB-0.11.0	0.0172	215	298	185	297	153	262	214	228	391
	MSHB-0.12.0	0.0172	215	298	197	293	153	266	240	228	391
	MSHB-0.13.0	0.0345	215	298	199	297	119	266	236	228	391
	MSHB-0.14.0	0.0172	215	298	201	287	119	266	220	ND	ND
	MSHB-0.15.0	0.0172	215	298	201	287	149	266	234	232	391
	MSHB-0.16.0	0.0172	215	298	201	295	119	262	214	228	391
	MSHB-0.17.0	0.0172	215	298	201	297	119	258	236	228	ND
	MSHB-0.18.0	0.0172	215	298	201	297	119	266	212	228	387
	MSHB-0.19.0	0.0172	215	298	201	297	119	266	234	228	391
	MSHB-0.20.0	0.0345	215	298	201	297	119	266	236	228	391

	MSHB-0.21.0	0.0172	215	298	201	297	119	266	246	232	391
	MSHB-0.22.0	0.0172	215	298	201	297	119	266	ND	230	391
	MSHB-0.23.0	0.0172	215	298	201	297	119	266	ND	234	391
	MSHB-0.24.0	0.0172	215	298	201	297	153	266	220	228	391
	MSHB-0.25.0	0.0172	215	298	201	297	ND	266	236	228	391
	MSHB-0.26.0	0.0172	215	298	201	297	ND	266	246	228	391
	MSHB-0.27.0	0.0172	235	290	199	297	119	266	232	228	391
	MSHB-0.28.0	0.0172	235	290	199	315	125	262	238	232	391
	MSHB-0.29.0	0.0172	235	298	201	315	125	266	238	232	391
	MSHB-0.30.0	0.0172	241	292	185	287	121	258	220	228	391
	MSHB-0.31.0	0.0172	241	292	185	287	121	266	218	230	ND
	MSHB-0.32.0	0.0172	241	292	185	287	149	258	220	228	ND
	MSHB-0.33.0	0.0172	241	292	185	287	149	266	212	242	391
	MSHB-0.34.0	0.0172	241	292	185	297	119	266	234	232	391
	MSHB-0.35.0	0.0172	241	292	185	297	149	266	220	ND	ND
	MSHB-0.36.0	0.0172	241	292	199	297	149	266	220	230	391
	MSHB-0.37.0	0.0172	241	292	201	297	119	266	246	228	391
	MSHB-0.38.0	0.0172	241	296	185	287	121	266	218	230	ND
	MSHB-0.39.0	0.0172	241	296	185	287	153	266	232	228	391
	MSHB-0.40.0	0.0172	241	296	197	293	121	266	234	244	391
	MSHB-0.41.0	0.0172	241	296	201	287	119	262	218	220	391
	MSHB-0.42.0	0.0172	241	296	201	297	119	266	220	230	391
	MSHB-0.43.0	0.0172	241	296	201	297	119	266	232	228	391
	MSHB-0.44.0	0.0172	241	298	185	287	ND	266	234	228	387
	MSHB-0.45.0	0.0172	241	298	201	297	121	266	234	228	391
Yunnan wild boar	MSYWB-0.1.0	0.0263	211	290	195	293	125	262	220	230	391
	MSYWB-0.2.0	0.0263	213	286	195	293	151	262	236	226	391
	MSYWB-0.3.0	0.0263	213	292	195	291	139	258	220	228	391
	MSYWB-0.4.0	0.0263	213	294	187	289	141	270	232	232	391
	MSYWB-0.5.0	0.0263	213	296	195	289	119	270	232	228	391
	MSYWB-0.6.0	0.0263	213	298	189	285	151	258	234	230	391
	MSYWB-0.7.0	0.0263	215	290	191	315	125	258	238	232	391
	MSYWB-0.8.0	0.0263	215	290	195	287	121	262	224	230	391
	MSYWB-0.9.0	0.0263	215	292	185	293	119	258	226	232	391
	MSYWB-0.10.0	0.0263	215	292	197	293	137	258	226	230	391
	MSYWB-0.11.0	0.0263	219	282	187	287	151	258	218	230	391
	MSYWB-0.12.0	0.0263	219	290	197	291	153	262	220	228	391
	MSYWB-0.13.0	0.0263	219	296	191	295	127	258	230	232	391
	MSYWB-0.14.0	0.0263	219	298	193	287	125	258	232	228	391
	MSYWB-0.15.0	0.0263	225	298	201	287	129	258	232	232	391
	MSYWB-0.16.0	0.0263	227	288	203	293	119	266	232	232	391
	MSYWB-0.17.0	0.0263	227	288	203	293	125	266	236	230	391
	MSYWB-0.18.0	0.0263	231	284	185	289	125	258	228	228	391
	MSYWB-0.19.0	0.0263	231	286	185	291	137	262	232	228	391
	MSYWB-0.20.0	0.0263	231	290	185	291	121	258	232	230	391
	MSYWB-0.21.0	0.0263	233	294	195	293	135	266	232	234	391
	MSYWB-0.22.0	0.0263	235	286	201	293	153	258	224	228	391
	MSYWB-0.23.0	0.0263	235	288	191	289	121	262	214	230	391
	MSYWB-0.24.0	0.0263	235	288	195	287	151	270	226	232	391
	MSYWB-0.25.0	0.0263	235	294	185	291	119	ND	236	230	391
	MSYWB-0.26.0	0.0263	235	294	185	295	141	266	226	234	391
	MSYWB-0.27.0	0.0263	235	294	195	289	129	266	234	230	391
	MSYWB-0.28.0	0.0263	237	288	195	291	129	258	220	228	391
	MSYWB-0.29.0	0.0263	237	294	187	289	133	266	230	232	391
	MSYWB-0.30.0	0.0263	237	296	195	289	133	ND	236	230	391
	MSYWB-0.31.0	0.0263	239	282	195	289	135	258	234	230	391
	MSYWB-0.32.0	0.0263	239	282	199	293	153	262	224	230	391
	MSYWB-0.33.0	0.0263	239	288	187	295	141	258	212	228	391

	MSYWB-0.34.0	0.0263	239	294	185	293	121	262	232	230	391
	MSYWB-0.35.0	0.0263	241	282	195	291	119	258	234	230	391
	MSYWB-0.36.0	0.0263	241	288	203	293	125	262	230	230	391
	MSYWB-0.37.0	0.0263	241	290	185	295	139	262	222	232	391
	MSYWB-0.38.0	0.0263	243	288	199	293	153	258	220	232	391
Huanan wild boar	MSHWB-0.1.0	0.0156	211	304	199	285	143	262	238	232	387
	MSHWB-0.2.0	0.0156	215	282	199	285	143	274	238	230	391
	MSHWB-0.3.0	0.0156	215	284	185	295	119	266	226	230	391
	MSHWB-0.4.0	0.0156	215	284	205	293	143	262	228	230	387
	MSHWB-0.5.0	0.0156	215	290	203	291	129	266	226	232	391
	MSHWB-0.6.0	0.0156	215	290	207	291	155	262	226	230	391
	MSHWB-0.7.0	0.0156	215	294	201	287	125	262	232	232	391
	MSHWB-0.8.0	0.0156	215	296	187	301	153	266	226	230	391
	MSHWB-0.9.0	0.0156	215	296	199	289	159	ND	238	230	391
	MSHWB-0.10.0	0.0156	215	298	187	301	155	274	238	230	391
	MSHWB-0.11.0	0.0156	215	298	193	287	157	262	218	226	395
	MSHWB-0.12.0	0.0156	215	298	201	287	155	262	218	228	391
	MSHWB-0.13.0	0.0156	215	298	201	293	149	262	220	226	395
	MSHWB-0.14.0	0.0156	215	298	201	295	135	262	236	236	391
	MSHWB-0.15.0	0.0156	215	300	187	301	151	262	236	228	391
	MSHWB-0.16.0	0.0156	215	304	207	295	131	262	236	226	391
	MSHWB-0.17.0	0.0156	215	ND	197	295	131	266	ND	240	391
	MSHWB-0.18.0	0.0156	231	282	195	289	153	266	238	230	391
	MSHWB-0.19.0	0.0156	231	ND	201	295	137	266	ND	228	391
	MSHWB-0.20.0	0.0156	233	284	195	293	137	266	238	232	391
	MSHWB-0.21.0	0.0156	233	284	201	285	135	266	220	230	391
	MSHWB-0.22.0	0.0156	233	284	201	295	159	274	230	240	391
	MSHWB-0.23.0	0.0156	233	292	197	295	137	262	236	232	395
	MSHWB-0.24.0	0.0156	233	292	201	295	135	ND	242	228	395
	MSHWB-0.25.0	0.0156	233	292	207	293	155	266	224	240	391
	MSHWB-0.26.0	0.0156	233	294	185	289	135	262	238	230	391
	MSHWB-0.27.0	0.0156	233	294	197	295	137	262	226	230	395
	MSHWB-0.28.0	0.0156	233	294	207	291	129	274	236	236	391
	MSHWB-0.29.0	0.0156	233	298	193	293	149	262	220	228	391
	MSHWB-0.30.0	0.0156	235	284	195	287	159	266	224	228	391
	MSHWB-0.31.0	0.0156	235	284	197	285	135	266	242	236	391
	MSHWB-0.32.0	0.0156	235	284	197	297	121	ND	242	ND	ND
	MSHWB-0.33.0	0.0156	235	284	199	285	159	262	228	230	395
	MSHWB-0.34.0	0.0156	235	286	199	295	133	262	220	230	391
	MSHWB-0.35.0	0.0156	235	288	199	287	153	266	220	240	391
	MSHWB-0.36.0	0.0156	235	290	209	301	155	274	236	228	391
	MSHWB-0.37.0	0.0156	235	292	195	293	125	262	236	228	391
	MSHWB-0.38.0	0.0156	235	292	201	295	135	266	236	240	ND
	MSHWB-0.39.0	0.0156	235	292	201	295	135	266	242	228	ND
	MSHWB-0.40.0	0.0156	235	294	201	285	119	266	238	234	391
	MSHWB-0.41.0	0.0156	235	304	199	293	137	ND	228	230	391
	MSHWB-0.42.0	0.0156	235	304	199	293	157	ND	226	226	391
	MSHWB-0.43.0	0.0156	235	304	205	293	143	262	238	230	ND
	MSHWB-0.44.0	0.0156	237	282	197	293	125	ND	220	230	ND
	MSHWB-0.45.0	0.0156	239	282	201	287	153	262	238	240	391
	MSHWB-0.46.0	0.0156	239	290	199	291	129	258	226	230	391
	MSHWB-0.47.0	0.0156	239	294	205	293	139	ND	228	240	391
	MSHWB-0.48.0	0.0156	241	282	197	293	125	ND	236	228	ND
	MSHWB-0.49.0	0.0156	241	282	205	295	143	262	228	230	391
	MSHWB-0.50.0	0.0156	241	284	197	291	137	262	236	232	391
	MSHWB-0.51.0	0.0156	241	284	199	285	119	262	228	234	ND
	MSHWB-0.52.0	0.0156	241	284	201	287	153	262	226	228	391
	MSHWB-0.53.0	0.0156	241	288	201	285	143	262	236	230	387

	MSHWB-0.54.0	0.0156	241	292	201	285	151	ND	226	ND	ND
	MSHWB-0.55.0	0.0156	241	292	201	291	159	266	226	228	391
	MSHWB-0.56.0	0.0156	241	292	201	295	155	274	226	242	391
	MSHWB-0.57.0	0.0156	241	294	187	301	137	262	218	232	391
	MSHWB-0.58.0	0.0156	241	294	195	295	125	ND	240	228	395
	MSHWB-0.59.0	0.0156	241	294	201	287	155	262	220	228	391
	MSHWB-0.60.0	0.0156	241	298	197	293	151	266	226	228	391
	MSHWB-0.61.0	0.0156	241	304	205	293	143	274	236	232	395
	MSHWB-0.62.0	0.0156	243	282	201	293	135	266	236	230	391
	MSHWB-0.63.0	0.0156	243	292	199	291	133	266	230	240	391
	MSHWB-0.64.0	0.0156	243	298	195	295	135	266	220	226	387
Dongbei wild boar	MSDWB-0.1.0	0.0167	211	294	195	293	125	274	220	232	391
	MSDWB-0.2.0	0.0156	211	294	201	293	135	262	220	232	ND
	MSDWB-0.3.0	0.0156	215	286	197	295	119	262	228	228	391
	MSDWB-0.4.0	0.0156	215	288	201	289	157	266	246	228	391
	MSDWB-0.5.0	0.0156	215	290	185	287	119	266	228	240	391
	MSDWB-0.6.0	0.0156	215	290	199	293	119	262	218	228	391
	MSDWB-0.7.0	0.0156	215	296	185	293	127	262	238	228	391
	MSDWB-0.8.0	0.0156	215	296	185	293	133	274	220	228	391
	MSDWB-0.9.0	0.0156	215	296	199	287	125	266	ND	244	391
	MSDWB-0.10.0	0.0156	215	296	201	295	119	262	232	232	391
	MSDWB-0.11.0	0.0156	215	296	201	295	125	262	232	230	391
	MSDWB-0.12.0	0.0156	215	296	201	295	125	274	238	232	391
	MSDWB-0.13.0	0.0313	215	296	201	295	141	262	234	232	391
	MSDWB-0.14.0	0.0469	215	296	201	295	141	262	238	232	391
	MSDWB-0.15.0	0.0156	215	296	201	295	141	274	238	232	391
	MSDWB-0.16.0	0.0156	215	298	185	293	133	262	ND	228	391
	MSDWB-0.17.0	0.0156	215	298	199	287	125	266	218	244	391
	MSDWB-0.18.0	0.0156	215	298	199	287	125	266	230	232	391
	MSDWB-0.19.0	0.0156	215	298	199	287	125	274	232	230	391
	MSDWB-0.20.0	0.0156	215	298	199	293	125	266	238	232	391
	MSDWB-0.21.0	0.0156	215	304	209	285	119	262	232	232	391
	MSDWB-0.22.0	0.0156	223	306	185	293	133	262	220	228	391
	MSDWB-0.23.0	0.0156	225	292	185	287	131	266	238	232	391
	MSDWB-0.24.0	0.0156	233	284	197	285	119	274	238	232	391
	MSDWB-0.25.0	0.0469	233	290	185	285	147	262	228	240	391
	MSDWB-0.26.0	0.0156	233	304	185	293	119	274	242	230	391
	MSDWB-0.27.0	0.0156	233	306	185	295	153	262	242	228	391
	MSDWB-0.28.0	0.0156	235	292	195	293	125	262	240	228	ND
	MSDWB-0.29.0	0.0156	235	292	201	287	119	266	220	242	391
	MSDWB-0.30.0	0.0156	237	284	209	295	125	266	226	228	387
	MSDWB-0.31.0	0.0156	237	296	185	295	119	262	218	230	387
	MSDWB-0.32.0	0.0156	241	282	197	285	119	262	238	230	387
	MSDWB-0.33.0	0.0156	241	288	185	293	125	274	222	230	391
	MSDWB-0.34.0	0.0156	241	292	185	285	153	266	220	240	391
	MSDWB-0.35.0	0.0313	241	292	185	287	131	266	226	230	391
	MSDWB-0.36.0	0.0167	241	292	185	287	131	266	226	232	391
	MSDWB-0.37.0	0.0156	241	292	185	287	131	266	236	232	391
	MSDWB-0.38.0	0.0313	241	292	185	287	153	266	220	230	391
	MSDWB-0.39.0	0.0156	241	292	185	295	133	266	236	228	391
	MSDWB-0.40.0	0.0156	241	294	185	ND	125	266	220	230	387
	MSDWB-0.41.0	0.0156	241	294	195	287	125	266	246	230	391
	MSDWB-0.42.0	0.0469	241	294	195	293	125	266	220	232	391
	MSDWB-0.43.0	0.0156	241	296	185	293	133	262	226	228	391
	MSDWB-0.44.0	0.0156	243	288	195	289	131	266	246	228	391
	MSDWB-0.45.0	0.0156	243	288	195	ND	125	266	244	232	387
	MSDWB-0.46.0	0.0156	243	294	195	289	125	274	238	228	391
	MSDWB-0.47.0	0.0156	243	296	185	295	141	262	234	230	391

#3 Rare haplotypes of SLA Class III region based on microsatellite typing (Frequency < 15%, ND: Not detected)

Population	Rare Haplotypes	Frequencies	Name of MS marker: SLAMS		
			056	098	A13
Diannan small-ear pig	MSDN-0.0.1	0.0334	274	329	203
	MSDN-0.0.2	0.0100	274	332	200
	MSDN-0.0.3	0.0568	274	332	203
	MSDN-0.0.4	0.0100	274	332	218
	MSDN-0.0.5	0.0100	274	332	227
	MSDN-0.0.6	0.0100	274	335	188
	MSDN-0.0.7	0.0890	274	335	200
	MSDN-0.0.8	0.0969	274	335	203
	MSDN-0.0.9	0.0600	274	335	206
	MSDN-0.0.10	0.0131	274	335	209
	MSDN-0.0.11	0.0100	274	335	215
	MSDN-0.0.12	0.0300	274	335	218
	MSDN-0.0.13	0.0153	274	335	224
	MSDN-0.0.14	0.0100	274	338	197
	MSDN-0.0.15	0.0375	274	338	200
	MSDN-0.0.16	0.0100	274	338	206
	MSDN-0.0.17	0.0569	274	338	209
	MSDN-0.0.18	0.0300	274	338	215
	MSDN-0.0.19	0.0292	274	338	224
	MSDN-0.0.20	0.0100	274	341	209
	MSDN-0.0.21	0.0166	276	329	203
	MSDN-0.0.22	0.0100	276	329	206
	MSDN-0.0.23	0.0144	276	332	203
	MSDN-0.0.24	0.0200	276	332	209
	MSDN-0.0.25	0.0188	276	332	224
	MSDN-0.0.26	0.0157	276	335	224
	MSDN-0.0.27	0.0235	276	338	200
	MSDN-0.0.28	0.0010	276	338	224
	MSDN-0.0.29	0.0100	ND	329	200
	MSDN-0.0.30	0.0100	ND	329	203
	MSDN-0.0.31	0.0100	ND	332	197
	MSDN-0.0.32	0.0100	ND	335	200
	MSDN-0.0.33	0.0100	ND	335	218
	MSDN-0.0.34	0.0100	ND	338	206
Congjiang Xiang pig	MSCJX-0.0.1	0.0429	274	338	203
	MSCJX-0.0.2	0.0286	274	338	209
	MSCJX-0.0.3	0.0143	274	338	230
	MSCJX-0.0.4	0.0143	276	329	203
	MSCJX-0.0.5	0.0143	276	329	206
	MSCJX-0.0.6	0.1189	276	335	203
	MSCJX-0.0.7	0.0143	276	335	206
	MSCJX-0.0.8	0.0143	276	335	224
	MSCJX-0.0.9	0.0143	276	335	230
	MSCJX-0.0.10	0.0286	276	338	188
	MSCJX-0.0.11	0.1143	276	338	206
	MSCJX-0.0.12	0.0143	276	338	230
Bama miniature pig	MSBM-0.0.1	0.0156	276	329	206
	MSBM-0.0.2	0.0168	276	332	203
	MSBM-0.0.3	0.0301	276	332	224
	MSBM-0.0.4	0.0156	276	332	230
	MSBM-0.0.5	0.0313	276	335	188
	MSBM-0.0.6	0.0469	276	335	200
	MSBM-0.0.7	0.1094	276	335	206
	MSBM-0.0.8	0.0172	276	335	209

	MSBM-0.0.9	0.0168	276	335	224
	MSBM-0.0.10	0.0313	276	335	236
	MSBM-0.0.11	0.1094	276	335	239
	MSBM-0.0.12	0.0485	276	338	203
	MSBM-0.0.13	0.0297	276	338	209
	MSBM-0.0.14	0.0156	278	332	239
	MSBM-0.0.15	0.0156	278	335	230
	MSBM-0.0.16	0.0313	278	338	206
Wuzhishan pig	MSWZS-0.0.1	0.0156	274	329	203
	MSWZS-0.0.2	0.0156	274	332	203
	MSWZS-0.0.3	0.0156	274	335	203
	MSWZS-0.0.4	0.0156	274	335	218
	MSWZS-0.0.5	0.0313	274	335	224
	MSWZS-0.0.6	0.0469	274	335	233
	MSWZS-0.0.7	0.0156	274	338	197
	MSWZS-0.0.8	0.0313	274	338	203
	MSWZS-0.0.9	0.0313	274	338	206
	MSWZS-0.0.10	0.0156	274	338	218
	MSWZS-0.0.11	0.0156	274	341	200
	MSWZS-0.0.12	0.0156	274	ND	200
	MSWZS-0.0.13	0.0469	274	ND	203
	MSWZS-0.0.14	0.0156	276	335	188
	MSWZS-0.0.15	0.0469	276	335	200
	MSWZS-0.0.16	0.0156	276	335	206
	MSWZS-0.0.17	0.0313	276	335	209
	MSWZS-0.0.18	0.0313	276	338	194
	MSWZS-0.0.19	0.0625	276	338	212
	MSWZS-0.0.20	0.0156	276	338	239
	MSWZS-0.0.21	0.0156	278	338	200
	MSWZS-0.0.22	0.0156	ND	335	200
	MSWZS-0.0.23	0.0156	ND	338	224
Mingguang small-ear pig	MSMG-0.0.1	0.0237	274	329	206
	MSMG-0.0.2	0.0100	274	329	215
	MSMG-0.0.3	0.0100	274	329	221
	MSMG-0.0.4	0.0200	274	329	224
	MSMG-0.0.5	0.0100	274	332	206
	MSMG-0.0.6	0.0200	274	332	215
	MSMG-0.0.7	0.0200	274	332	221
	MSMG-0.0.8	0.0100	274	332	224
	MSMG-0.0.9	0.0200	274	332	227
	MSMG-0.0.10	0.0828	274	335	200
	MSMG-0.0.11	0.0800	274	335	203
	MSMG-0.0.12	0.0756	274	335	206
	MSMG-0.0.13	0.0375	274	335	209
	MSMG-0.0.14	0.0100	274	335	212
	MSMG-0.0.15	0.0300	274	335	215
	MSMG-0.0.16	0.0115	274	335	224
	MSMG-0.0.17	0.0485	274	335	227
	MSMG-0.0.18	0.0100	274	338	200
	MSMG-0.0.19	0.0353	274	338	203
	MSMG-0.0.20	0.0351	274	338	206
	MSMG-0.0.21	0.0300	274	338	215
	MSMG-0.0.22	0.0200	274	338	221
	MSMG-0.0.23	0.0100	274	338	227
	MSMG-0.0.24	0.0100	274	341	203
	MSMG-0.0.25	0.0100	274	341	206
	MSMG-0.0.26	0.0200	274	ND	206
	MSMG-0.0.27	0.0163	276	329	200

	MSMG-0.0.28	0.0100	276	329	239
	MSMG-0.0.29	0.0100	276	332	200
	MSMG-0.0.30	0.0100	276	332	206
	MSMG-0.0.31	0.0309	276	335	200
	MSMG-0.0.32	0.0356	276	335	206
	MSMG-0.0.33	0.0125	276	335	209
	MSMG-0.0.34	0.0300	276	335	221
	MSMG-0.0.35	0.0336	276	335	224
	MSMG-0.0.36	0.0115	276	335	227
	MSMG-0.0.37	0.0347	276	338	203
	MSMG-0.0.38	0.0100	276	338	212
	MSMG-0.0.39	0.0149	276	338	224
	MSMG-0.0.40	0.0100	276	ND	203
	MSMG-0.0.41	0.0100	276	ND	212
	MSMG-0.0.42	0.0100	ND	335	218
	MSMG-0.0.43	0.0100	ND	335	224
Hezuo pig	MSHZ-0.0.1	0.0143	266	335	218
	MSHZ-0.0.2	0.0698	276	329	203
	MSHZ-0.0.3	0.0159	276	329	212
	MSHZ-0.0.4	0.0143	276	329	227
	MSHZ-0.0.5	0.0143	276	329	239
	MSHZ-0.0.6	0.1002	276	332	203
	MSHZ-0.0.7	0.0143	276	335	206
	MSHZ-0.0.8	0.0166	276	335	212
	MSHZ-0.0.9	0.0429	276	335	218
	MSHZ-0.0.10	0.0400	276	338	200
	MSHZ-0.0.11	0.0374	276	338	203
	MSHZ-0.0.12	0.0286	276	338	206
	MSHZ-0.0.13	0.0675	276	338	212
	MSHZ-0.0.14	0.0190	276	338	227
	MSHZ-	0.0570	278	332	203
	MSHZ-0.0.16	0.0478	278	335	203
	MSHZ-0.0.17	0.0143	278	335	218
	MSHZ-0.0.18	0.0143	278	335	224
	MSHZ-0.0.19	0.0164	278	335	227
	MSHZ-0.0.20	0.0217	278	338	227
Hebao pig	MSHB-0.0.1	0.0212	276	332	200
	MSHB-0.0.2	0.0172	276	332	224
	MSHB-0.0.3	0.0172	276	335	224
	MSHB-0.0.4	0.0172	276	335	239
	MSHB-0.0.5	0.0345	278	332	200
	MSHB-0.0.6	0.0690	278	335	200
	MSHB-0.0.7	0.0172	278	338	200
	MSHB-0.0.8	0.0172	282	332	227
Yunnan wild boar	MSYWB-0.0.1	0.0526	274	329	200
	MSYWB-0.0.2	0.0263	274	332	203
	MSYWB-0.0.3	0.0869	274	335	203
	MSYWB-0.0.4	0.0526	274	335	206
	MSYWB-0.0.5	0.0974	274	335	209
	MSYWB-0.0.6	0.0526	274	338	203
	MSYWB-0.0.7	0.0526	274	338	224
	MSYWB-0.0.8	0.0526	274	341	200
	MSYWB-0.0.9	0.0263	276	332	194
	MSYWB-0.0.10	0.0263	276	332	200
	MSYWB-0.0.11	0.0263	276	332	206
	MSYWB-0.0.12	0.0263	276	335	200
	MSYWB-0.0.13	0.0710	276	335	203
	MSYWB-0.0.14	0.0342	276	335	209

	MSYWB-0.0.15	0.0526	ND	335	203
	MSYWB-0.0.16	0.0526	ND	338	200
	MSYWB-0.0.17	0.0263	ND	338	203
	MSYWB-0.0.18	0.0263	ND	338	206
Huanan wild boar	MSHWB-0.0.1	0.0156	274	335	194
	MSHWB-0.0.2	0.1330	274	335	200
	MSHWB-0.0.3	0.0237	274	335	203
	MSHWB-0.0.4	0.0313	274	335	206
	MSHWB-0.0.5	0.0621	274	335	209
	MSHWB-0.0.6	0.0469	274	338	188
	MSHWB-0.0.7	0.0156	274	338	197
	MSHWB-0.0.8	0.0819	274	338	200
	MSHWB-0.0.9	0.1013	274	338	203
	MSHWB-0.0.10	0.0523	274	338	206
	MSHWB-0.0.11	0.0927	274	338	209
	MSHWB-0.0.12	0.0156	274	338	218
	MSHWB-0.0.13	0.0156	274	338	230
	MSHWB-0.0.14	0.0156	274	ND	200
	MSHWB-0.0.15	0.0232	276	335	200
	MSHWB-0.0.16	0.0221	276	335	206
	MSHWB-0.0.17	0.0328	276	335	209
	MSHWB-0.0.18	0.1056	276	338	200
	MSHWB-0.0.19	0.0194	276	338	206
	MSHWB-0.0.20	0.0156	276	ND	218
	MSHWB-0.0.21	0.0156	278	338	203
	MSHWB-0.0.22	0.0156	ND	335	203
	MSHWB-0.0.23	0.0156	ND	335	206
	MSHWB-0.0.24	0.0156	ND	338	200
	MSHWB-0.0.25	0.0156	ND	338	209
Dongbei wild boar	MSDWB-0.0.1	0.0313	276	332	227
	MSDWB-0.0.2	0.1423	276	335	200
	MSDWB-0.0.3	0.1234	276	335	203
	MSDWB-0.0.4	0.0469	276	335	209
	MSDWB-0.0.5	0.0156	276	335	221
	MSDWB-0.0.6	0.0156	278	335	224
	MSDWB-0.0.7	0.0781	278	338	203
